# Test-retest reproducibility of quantitative binding measures of [^11^C]Ro15-4513, a PET ligand for GABA_A_ receptors containing alpha5 subunits

**DOI:** 10.1016/j.neuroimage.2016.12.038

**Published:** 2017-05-15

**Authors:** Colm J. McGinnity, Daniela A. Riaño Barros, Lula Rosso, Mattia Veronese, Gaia Rizzo, Alessandra Bertoldo, Rainer Hinz, Federico E. Turkheimer, Matthias J. Koepp, Alexander Hammers

**Affiliations:** a Centre for Neuroscience, Department of Medicine, Imperial College London, London, UK; b Medical Research Council Clinical Sciences Centre, Hammersmith Hospital, London, UK; c Division of Imaging Sciences & Biomedical Engineering, King’s College London, London, UK; d Centre for Neuroimaging Sciences, Institute of Psychiatry, Psychology & Neuroscience, King's College London, London, UK; e Department of Information Engineering, University of Padova, Padova, Italy; f Wolfson Molecular Imaging Centre, University of Manchester, Manchester, UK; g Department of Clinical and Experimental Epilepsy, Institute of Neurology, University College London, UK; h Epilepsy Society, Chalfont St Peter, UK; i The Neurodis Foundation, CERMEP - Imagerie du Vivant, Lyon, France

**Keywords:** BP_ND_, binding potential relative to non-displaceable binding, BS-CV, between-subject coefficient of variation, FBP, filtered backprojection, FORE, Fourier rebinning, GABA_A_, γ-aminobutyric acid “A” receptor, ICC, intraclass correlation coefficient, MAPER, multi-atlas propagation with enhanced registration, NHS, National Health Service (of the United Kingdom), MA-TD, median absolute percentage test – retest difference, NNLS, non-negative least squares, ppIFs, metabolite-corrected, arterial parent plasma input functions, ROI, region-of-interest, RPM, Receptor Parametric Mapping, SA, (exponential) spectral analysis, SRTM, simplified reference tissue model, SUV, standardised uptake values, TACs, time-activity curves, V_T_, (total) volume-of-distribution, WS-CV, within-subject coefficient of variation, GABA_A_, α5, Alpha5, Positron emission tomography, Reliability, Reproducibility

## Abstract

**Introduction:**

Alteration of γ-aminobutyric acid “A” (GABA_A_) receptor-mediated neurotransmission has been associated with various neurological and psychiatric disorders. [^11^C]Ro15-4513 is a PET ligand with high affinity for α5-subunit-containing GABA_A_ receptors, which are highly expressed in limbic regions of the human brain (Sur et al., 1998). We quantified the test-retest reproducibility of measures of [^11^C]Ro15-4513 binding derived from six different quantification methods (12 variants).

**Methods:**

Five healthy males (median age 40 years, range 38–49 years) had a 90-min PET scan on two occasions (median interval 12 days, range 11–30 days), after injection of a median dose of 441 MegaBequerels of [^11^C]Ro15-4513. Metabolite-corrected arterial plasma input functions (parent plasma input functions, ppIFs) were generated for all scans.

We quantified regional binding using six methods (12 variants), some of which were region-based (applied to the average time-activity curve within a region) and others were voxel-based: 1) Models requiring arterial ppIFs – regional reversible compartmental models with one and two tissue compartments (2kbv and 4kbv); 2) Regional and voxelwise Logan’s graphical analyses (Logan et al., 1990), which required arterial ppIFs; 3) Model-free regional and voxelwise (exponential) spectral analyses (SA; (Cunningham and Jones, 1993)), which also required arterial ppIFs; 4) methods not requiring arterial ppIFs – voxelwise standardised uptake values (Kenney et al., 1941), and regional and voxelwise simplified reference tissue models (SRTM/SRTM2) using brainstem or alternatively cerebellum as pseudo-reference regions (Lammertsma and Hume, 1996; Gunn et al., 1997).

To compare the variants, we sampled the mean values of the outcome parameters within six bilateral, non-reference grey matter regions-of-interest. Reliability was quantified in terms of median absolute percentage test-retest differences (MA-TDs; preferentially low) and between-subject coefficient of variation (BS-CV, preferentially high), both compounded by the intraclass correlation coefficient (ICC). These measures were compared between variants, with particular interest in the hippocampus.

**Results:**

Two of the six methods (5/12 variants) yielded reproducible data (i.e. MA-TD <10%): regional SRTMs and voxelwise SRTM2s, both using either the brainstem or the cerebellum; and voxelwise SA. However, the SRTMs using the brainstem yielded a lower median BS-CV (7% for regional, 7% voxelwise) than the other variants (8–11%), resulting in lower ICCs. The median ICCs across six regions were 0.89 (interquartile range 0.75–0.90) for voxelwise SA, 0.71 (0.64–0.84) for regional SRTM-cerebellum and 0.83 (0.70–0.86) for voxelwise SRTM-cerebellum. The ICCs for the hippocampus were 0.89 for voxelwise SA, 0.95 for regional SRTM-cerebellum and 0.93 for voxelwise SRTM-cerebellum.

**Conclusion:**

Quantification of [^11^C]Ro15-4513 binding shows very good to excellent reproducibility with SRTM and with voxelwise SA which, however, requires an arterial ppIF. Quantification in the α5 subunit-rich hippocampus is particularly reliable. The very low expression of the α5 in the cerebellum (Fritschy and Mohler, 1995; Veronese et al., 2016) and the substantial α1 subunit density in this region may hamper the application of reference tissue methods.

## 1 Introduction

γ-aminobutyric acid (GABA) is one of the principal inhibitory neurotransmitters in the brain (Curtis et al., 1970). The ligand-gated, ion channel GABA_A_ receptor (Lobo and Harris, 2008) is a pentameric structure assembled from five of 19 known protein subunits: α_1–6_, β_1–3_, γ_1–3_, δ, ε, π, ρ_1–3_ and θ (Barnard et al., 1998). On binding GABA, the permeability of the central pore to chloride ions increases, resulting in hyperpolarisation of the neuron and hence reduced excitability. The receptor subunits exhibit distinct but overlapping distributions within the brain (Fritschy and Mohler, 1995, Sieghart and Sperk, 2002) and roles that change during development and with pathologies (Galanopoulou, 2008).

Approximately 5% of GABA_A_ receptors contain the α5 subunit. The hippocampus is the structure with the highest concentration of α5-subunit-containing receptors in the human brain; for example, an ex vivo study with the α5-subunit-selective radioligand [^3^H]L-655,708 suggested they are present in almost 28% of GABA_A_ receptors in this region (Sur et al., 1998). Here, they have a predominantly extra-synaptic localisation (Brunig et al., 2002) and mediate tonic inhibitory currents (Caraiscos et al., 2004). Experiments in animals suggest that agonists at receptors containing the α5 subunit negatively influence hippocampus-dependent learning and memory (Collinson et al., 2002, Crestani et al., 2002, Sternfeld et al., 2004, Yee et al., 2004, Cheng et al., 2006, Dawson et al., 2006). Whilst data in humans is lacking, the amnestic effect of alcohol on wordlist learning was reduced by pre-treatment with an α5-subunit-selective inverse agonist (Nutt et al., 2007).

Alteration of GABA_A_ receptor-mediated neurotransmission has been associated with a wide variety of neurological and psychiatric disorders (Korpi and Sinkkonen, 2006), including alcohol dependence, anxiety (reviewed by Roy-Byrne (2005)), epilepsy ((Macdonald et al., 2004), reviewed by Gonzalez and Brooks-Kayal (2011)), and schizophrenia (reviewed by Charych et al. (2009)).

The synthesis of subtype-selective tracers such as Ro15-4513 (ethyl-8-azido-5,6-dihydro-5-methyl-6-oxo-4H-imidazo-1,4-benzodiazepine-3-carboxylate; F. Hoffmann–La Roche Ltd., Basel, Switzerland) promises further understanding of GABA_A_ receptors. Ro15-4513 is an α5-subunit-selective imidazodiazepine that behaves as a partial inverse agonist at GABA_A_ receptors (Bonetti et al., 1988, Lister and Nutt, 1988, Sadzot et al., 1989). Receptors that express α5 subunits have 10–15-fold higher affinity to Ro15-4513 than those that do not (Hadingham et al., 1993, Luddens et al., 1994). Competition studies in the rat in vivo revealed that radiolabelled Ro15-4513 binding was reduced to nonspecific levels only by drugs that have affinity for the α5 subtype (flunitrazepam, RY80, Ro15-4513, L655,708) and that [^11^C]Ro15-4513 has a tenfold higher affinity to α5 than α1 receptors (Lingford-Hughes et al., 2002).

In healthy human volunteers, pre-scan administration of the α1 antagonist zolpidem did not significantly decrease *total* [^11^C]Ro15-4513 volume-of-distribution (V_T_; (Myers et al., 2012))_,_ but alteration of the fast (α1) component peaks (derived using exponential spectral analysis; SA) was observed. In the α5-rich hippocampus, the mean volume-of-distribution attributable to the fast peaks was reduced by approximately 70% to 0.44, whereas a slower component (presumably attributable to the a5 subunit) was reduced by approximately 13% to 10.00. More recently, human heterologous competition data acquired from nine healthy males using the α5-subunit-selective negative allosteric modulator, Basmisanil (RG1662), suggested that α5-specific binding accounts for 76% of the specific binding in the hippocampus (Myers et al., 2016).

Inoue et al. (1992) used the simplified reference tissue model (SRTM) with pons as a (pseudo-) reference region to demonstrate the differences between the distribution of [^11^C]Ro15-4513 and [^11^C]flumazenil in five healthy participants. [^11^C]Ro15-4513 V_T_ has also been previously quantified by voxel-wise exponential SA in three healthy males (Lingford-Hughes et al., 2002). Comparison with [^11^C]flumazenil PET derived from six healthy males indicated significantly greater V_T_ (by approximately 36%) in the hippocampus for [^11^C]Ro15-4513, with significantly less binding (approximately 43%) in the cerebellum.

[^11^C]Ro15-4513 binding has also been quantified in eight healthy men using both compartmental modelling and linear graphical analyses (Asai et al., 2009); the SRTM with pons as a reference was recommended based on resilience to noise, but test – retest studies were not performed.

[^11^C]Ro15-4513 PET has recently been used to demonstrate alterations in GABA_A_ α5 subunit binding in alcohol dependence (Lingford-Hughes et al., 2012), schizophrenia (Asai et al., 2008), in response to levodopa challenge (Lou et al., 2016), and in preliminary studies in temporal lobe epilepsy (Barros et al., 2010a) and autism spectrum disorder (Mendez et al., 2013). To facilitate interpretation, it is necessary to document the reproducibility and reliability of quantitative methods, both regional and voxelwise, for [^11^C]Ro15-4513 PET. Whilst the test – retest variability of presumed α1- and α5-subunit-specific V_T_ has been reported (Stokes et al., 2014), the variability of total V_T_ has not. Moreover, the analysis performed by Stokes and colleagues was restricted to a single quantification method (SA), whereas variants that do not require an arterial input function, and variants which yield parametric images, still merit investigation. However, no such analysis has been published.

In the present study we quantified the test-retest reproducibility and reliability of measures of [^11^C]Ro15-4513 binding derived from six quantification methods, namely standardised uptake values (SUVs), one tissue compartment and two tissue compartment models (2kbv and 4kbv), graphical (linear) analyses, SA, and SRTMs, in regions representative of a variety of α5 subunit concentrations, in five healthy volunteers.

## 2 Materials and methods

### 2.1 Participants

The study was approved by the London – Riverside National Health Service (NHS) Research Ethics Committee, Imperial College Healthcare NHS Trust, and University College London Hospitals NHS Foundation Trust. Permission to administer [^11^C]Ro15-4513 was obtained from the Administration of Radioactive Substances Advisory Committee, UK. All participants provided written, informed consent according to the Declaration of Helsinki prior to participation in the study.

Seven healthy male participants were recruited and provided written, informed consent. The exclusion criteria were: a history of either neurological or psychiatric condition(s), claustrophobia, any contraindication for undergoing magnetic resonance (MR) imaging, a positive urine drug test, general practitioner’s (family doctor’s) advice against participation, regular medication(s) (especially benzodiazepines), a history of substance abuse (especially benzodiazepines), and a pathological modified Allen’s test for patency of the ulnar artery (Allen, 1929, Slogoff et al., 1983, Cable et al., 1999). Two of the seven participants were subsequently excluded: one who withdrew from the study before the second scan, and another in whom the arterial line could not be kept patent for the entire study. Hence, a total of five healthy male participants (median age 40 years, range 38–49 years; Table 1) were scanned twice. All participants underwent a urine drug cassette test for 11-nor-Δ^9^-tetrahydrocannabinol, morphine, amphetamine, benzoylecgonine (the main metabolite of cocaine), methamphetamine and oxazepam (Monitect; BMC, California, USA) on the same day as each PET scan.

**Table 1:** Demographic and injectate details for the participants.

**Participant no.**	**Age**	**Gender**	**BMI**	**Interval (days)**	**Dose (MBq)**	**Radiochemical purity (%)**	**Co-injected mass (μg)**	**Specific Radioactivity (MBq/nmol)**
1	40	M	25	12	431	99	2.0	72
					435	98	2.4	59
2	38	M	27	30	430	99	4.4	32
					437	98	2.2	66
3	39	M	20	11	447	99	3.0	49
					441	99	5.1	28
4	49	M	34	59	440	97	2.6	55
					442	98	3.5	42
5	44	M	23	7	452	96	3.0	26
					444	98	0.4	72
**Median**	40		25	12	441	98	2.8	52
**Interquartile range (25**th**–75**th perc**)**	39–44		23–27	11–30	435–443	98–99	2.2–3.4	34–64
**Min**	38		20	7	430	96	0.4	26
**Max**	49		34	59	452	99	5.1	72

BMI=Body mass index; MBq=MegaBequerels; Min=Minimum; Max=Maximum; μg=Micrograms; nmol=Nanomoles; no=Number; perc=Percentiles.

### 2.2 Radiochemistry

[^11^C]Ro15-4513 was produced on site by Hammersmith Imanet as described previously (Myers et al., 2012). Details of the injectate are provided in Table 1. Specific radioactivities at the time of injection were calculated in relation to the relative molecular weight of Ro15-4513 (326 mol/g).

### 2.3 PET data acquisition

PET scans were acquired on a Siemens/CTI ECAT EXACT HR+(“962”) camera (Knoxville, TN, USA; (Spinks et al., 2000)) in 3D mode. Ten-minute transmission scans for attenuation and scatter correction were obtained prior to the dynamic emission scans using a rotating ^68^Ge rod source. Each dynamic acquisition was 90.5 min long and consisted of 24 frames of increasing length (1×30 s (s), 4×15 s, 4×60 s, 2×150 s, 10×300 s, 3×600 s). [^11^C]Ro15-4513 was injected as an intravenous bolus injection of ~440 MegaBequerels (MBq; median 441 MBq, range 430–452 MBq; Table 1) at 30 s after the dynamic scan start. Participants were scanned on two separate days with a median interval of 12 days (range 7 – 59 days; Table 1).

The head position was maintained throughout and monitored with the camera’s positioning laser. To compensate for minor head movements during the dynamic scans, we used a post hoc frame-by-frame realignment method, as described later (“PET data quantification” section). Sixty-three transaxial images were acquired per frame. Data were reconstructed using Fourier rebinning (FORE; (Defrise et al., 1997)) and 2D filtered backprojection (FBP: ramp filter, kernel 2.0 millimetres (mm) Full Width at Half Maximum (FWHM)). The voxel size of reconstructed images was 2.092 mm×2.092 mm×2.42 mm and the axial (on-axis) resolution 5.6 mm (Spinks et al., 2000).

### 2.4 Input function derivation

As in previous studies, continuous and intermittent blood samples were collected to allow the subsequent generation of arterial parent plasma input functions (ppIFs; (Hammers et al., 2008; Riaño Barros et al., 2014)). During the first 15 min, blood was withdrawn continuously at a rate of 300 millilitres (ml) per hour and radioactivity detected in a bismuth germanium oxide detection system (Ranicar et al., 1991). Intermittent discrete (10 ml) samples were taken using heparinised syringes before the scan (baseline) and at the following time points after the scan start: 4, 6, 8, 10, 20, 35, 50, 65, 80 and 90 min. These discrete samples were used to quantify plasma and whole blood radioactivity via centrifugation, as well as to allow quantification of the parent fraction of the radiotracer via high-performance liquid chromatography. The plasma-over-blood ratio model and the metabolite model were fit for each scan, so that the whole blood measurements between 0 and 15 min could be corrected for plasma-over-blood ratio and parent fraction, respectively. The plasma-over-blood ratio model used was as follows:

(1) $$1\;-\;\frac{x_{1}+x_{2}\cdot t_{s}}{{(x_{3}/t_{s})}^{x_{4}}+1}$$

[where t_s_ is the time from scan start in hours and x_1_ to x_4_ are the model parameters]. The same sigmoidal model function was used to describe the fraction of parent [^11^C]Ro15-4513 remaining in plasma, as in (Myers et al., 2016).

Continuous ppIFs were derived using Clickfit in-house software running in MATLAB R2014a (The MathWorks, Natick, MA, USA), as described in detail in previous studies (Hammers et al., 2007b; Hinz et al., 2007):

1) Cross-calibration of continuous and discrete whole blood radioactivity concentration measurements (4, 6, 8, 10 min).
2) Multiplication of the cross-calibrated whole blood measurements (0–15 min) by the sigmoid function obtained from fitting the model to the plasma-over blood ratio.
3) Combination of the resultant continuous plasma radioactivity concentration curve (0–15 min) with the discrete plasma radioactivity concentration measurements (20, 35, 50, 65, 80 and 90 min) using spline interpolation.
4) Correction for parent radiotracer fraction by multiplication of the resultant continuous plasma radioactivity concentration curve (0–90 min) by the sigmoid function obtained from fitting the model to the parent fraction.

### 2.5 MRI data acquisition, analysis and generation of regions-of-interest (ROIs)

All participants had 3D T1-weighted MRI scans with approximately millimetric isotropic voxel sizes on a Philips Intera 3 Tesla (3 T) MRI scanner (Philips, Best, The Netherlands) at the Robert Steiner MRI Unit, Hammersmith Hospital, for use during co-registration and region-of-interest (ROI) delineation. There was no gross structural abnormality on any of the T1-weighted images.

To isolate the grey matter in each brain, the T1-weighted images were segmented into tissue classes using SPM8 software (Statistical Parametric Mapping, Wellcome Trust Centre for Neuroimaging, University College London, London, www.fil.ion.ucl.ac.uk/spm) running in MATLAB R2014a. This process yielded grey matter probability maps for each participant, which were thresholded at 0.5 probability (an arbitrary value which was selected as a trade-off between over-exclusion of grey matter and over-inclusion of white matter).

To delineate the ROIs in each brain, the T1-weighted images were also anatomically segmented using MAPER (multi-atlas propagation with enhanced registration (Heckemann et al., 2010)). Using high-dimensional image registration, 30 MRI data sets, each associated with manually determined labels of 83 regions (Hammers et al., 2003, Gousias et al., 2008), were propagated to the target brain. Label fusion was used to obtain an image which consisted of 83 labelled ROIs in target space (Heckemann et al., 2006).

Each participant’s T1-weighted image and corresponding MAPER-derived individual anatomical segmentations, as well as individual grey matter probability images, were co-registered with the corresponding processed PET summation image for test and retest scans separately, using SPM8. For the cortical ROIs, the individual atlases in PET space were then multiplied with the thresholded grey matter probability maps using Analyze 8.1 imaging software (Mayo Clinic 2002). The output grey-matter-masked, labelled ROI images were then used to sample the dynamic or parametric PET images.

### 2.6 ROIs

We evaluated test – retest reliability of the quantification methods (12 variants) in six bilateral ROIs in total. Based on the expected concentrations of GABA_A_ receptors containing α5 subunits, we selected ROIs to cover a range of binding: high-concentration limbic regions – anterior cingulate gyrus and hippocampus; intermediate concentration regions – fusiform (occipitotemporal) gyrus, inferior frontal gyrus and insula; and a low concentration region – the occipital lobes. The (entire) brainstem and alternatively the grey matter of the cerebellum were used as pseudo-reference regions for the SRTMs. Here, the term “pseudo-reference” region is used as neither the brainstem nor the cerebellum are entirely devoid of α5 subunit specific binding. The methods not relying on a reference region as input were also applied to these regions, but comparison between variants was limited to the six non-reference ROIs only. Regional time-activity curves (TACs) were created by calculation of the mean radioactivity concentration over all grey-matter masked voxels of both left and right hemisphere homologues (excluding the brainstem which is not paired and was not grey-matter masked), for each frame. Likewise, where parametric images were used, the mean was calculated over all voxels of both homologues (again excluding the brainstem).

### 2.7 PET data quantification

Where required (one of 10 datasets, based on a maximum translation >5 mm as estimated using SPM “Realign” function), the dynamic PET images were de-noised and corrected for movement frame-by-frame using wavelets in Piwave 8.0 (Studholme et al., 1997, Turkheimer et al., 1999) running in MATLAB R2014a. The frame starting at 150 s (frame 6) was used as reference due to its high signal-to-noise ratio and the likelihood that the participant had remained still during the first three minutes of the scan. The frames 1–5 were not corrected due to their low signal to noise ratio. The remaining frames (6–24) were automatically resliced and re-concatenated with frames 1–5 into a new dynamic image (Hammers et al., 2007b).

Summation images (also known as ‘add’ or ‘static’ images) were created for frames 1–24, frames 16–21, and frames 22–24 with correction for ^11^C radiodecay using MICKPM (Modelling, Input functions, and Compartmental Kinetics – Parametric Maps) version 5.4 software (available on request from Rainer Hinz, Wolfson Molecular Imaging Centre, University of Manchester, Manchester, UK), which itself uses MATLAB R2009bSP1. The summation images were required for calculation of global radioactivity concentrations, for use as the reference image during co-registration of the T1-weighted MRI data, and as the input for calculation of SUV images. A binary mask of the brain, which encompassed approximately 9 mm beyond the outer cortical boundary, was also created semi-automatically using Analyze 8.1 (Mayo Clinic, Rochester, New York) for use during the subsequent generation of parametric images.

Quantification of binding was performed as described below, using the same ROIs for each variant. We used a 64-bit PC (Intel Core i5 5300U CPU 2.30 GHz, 8.0 GB RAM) with Windows 7 Enterprise operating system. Variants in which parameters were calculated directly from the ROI TAC data (generated as described in Section 2.6) are henceforth referred to as “regional”. Variants in which parameters were calculated on a voxel-by-voxel basis in order to generate parametric images are referred to as “voxelwise”; in this case the parametric image itself was sampled (mean, standard deviation) within same ROIs as used for regional variants. The quantification methods were:

1. Compartmental models, requiring arterial ppIFs
  (a) Reversible two-compartment (one tissue compartment) model with two rate constants and variable blood volume (2kbv)
  (b) Reversible three-compartment (two tissue compartment) model with four rate constants and variable blood volume (4kbv)
2. Graphical analyses, requiring arterial ppIFs
  (a) Regional Logan’s graphical analysis with arterial ppIF
  (b) Voxelwise Logan’s graphical analysis with arterial ppIF
3. Model-free analyses, requiring arterial ppIFs
  (a) Regional “classic” (non-regularised) SA
  (b) Voxelwise “classic” SA
4. Methods not requiring arterial ppIFs
  (a) Voxelwise standardised uptake values (SUVs)
    (i) 30.5–60.5 min
    (ii) 60.5–90.5 min
  (b) Regional SRTM using brainstem
  (c) Voxelwise SRTM2 using brainstem
  (d) Regional SRTM using cerebellum
  (e) Voxelwise SRTM2 using cerebellum

### 2.8 Weighting of ROI and voxel TACs

For each participant, all ROI/voxel TACs (not yet corrected for decay-correction) were weighted by the same values which were calculated according to:

(2) $$w_{i}=\frac{L_{i}}{T_{i}}\;\mathrm{for}\;\mathrm{frame}\;(i\;=\;1,\;2,\;3,\;\ldots 24;\;\mathrm{non}-\mathrm{decay}\;\mathrm{corrected}\;\mathrm{data})$$

[where W_i_ – weight for frame i; L_i_ – length of frame i (seconds); T_i_ – total of true coincidences (per second) for frame i].

For ROI TACs, the weights were normalised to sum(weights)=24 (i.e. number of frames), thresholded to max(weight)≤2.5, and then re-normalised to sum(weights)=24. For voxel TACs, the weights were not normalised but were thresholded to max(weight)/min(weight)≤1000, according to the RPM (Receptor Parametric Mapping software) scheme (Gunn et al., 1998, Aston et al., 2001).

### 2.9 Reversible compartmental models, requiring arterial ppIFs

MICK (Modelling, Input functions and Compartmental Kinetics) version 5.2 software (available on request from Rainer Hinz, Wolfson Molecular Imaging Centre, University of Manchester, Manchester, UK; see Supplementary material) was used to fit all regional compartmental models with the Nelder-Mead optimisation algorithm (Nelder and Mead, 1965). MICK uses MATLAB R2009bSP1.

#### 2.9.1 2kbv

In this model, three microparameters are derived: K1, rate constant for influx of the ligand from the plasma to the tissue compartment containing free, non-specifically bound, and specifically bound ligand; k2, efflux rate constant from the tissue back to plasma; and bv, the blood volume term. The V_T_ is then calculated according to the compartmental model equation (Innis et al., 2007).

(3) $$V_{T}=\frac{K_{1}}{k_{2}}$$

We used starting estimates of K1=0.01 ml cm^-3^ min^-1^, k2=0.001 min^-1^ and bv=0.05.

#### 2.9.2 4kbv

In addition to K1 and k2 (described above for the 2kbv model), two further rate constants were estimated: k3, which describes the transfer from the free and non-specifically bound compartment to the specifically bound (second tissue) compartment; and k4, which describes the opposite transfer. Again, the blood volume term was also computed. According to the consensus nomenclature (Innis et al., 2007):

(4) $$V_{T}=\frac{K_{1}}{k_{2}}(1+\frac{k_{3}}{k_{4}})$$

We used starting estimates of K1=0.01 ml cm^-3^ min^-1^, k2–k4=0.001 min^-1^ and bv=0.05.

### 2.10 Graphical analyses, requiring arterial ppIFs

#### 2.10.1 Regional Logan’s graphical analysis with arterial ppIF

Logan’s graphical analysis (Logan et al., 1990) is a linear analysis applicable to radioligands with reversible binding. After some time (t*), a plot of

(5) $$\frac{\int_{0}^{t}\mathrm{TAC}\left(t^{\prime }\right)\mathrm{dt}\prime }{\mathrm{TAC}(t)}\mathrm{versus}\frac{\int_{0}^{t}\mathrm{ppIF}\left(t^{\prime }\right)\mathrm{dt}\prime }{\mathrm{TAC}(t)}$$

is linear; where ppIF(t) – metabolite-corrected plasma radioactivity concentration at time t, TAC(t) – region of interest radioactivity concentration at time t. For the two-tissue compartment model 4kbv, the slope of the plot is:

(6) $$\frac{K_{1}}{k_{2}}(1+\frac{k_{3}}{k_{4}})+\mathrm{bv}$$

[where bv is the blood volume term], from which V_T_ can be calculated as above. MICK was used to fit regional Logan’s graphical analyses with the Nelder-Mead optimisation algorithm (Nelder and Mead, 1965). We used fixed parameters of t*=1680 s (i.e. 28 min, based on a preliminary inspection of the plots), bv=0.028 (the median across 83 brain regions and the 10 scans, as estimated using 4kbv; interquartile range 0.022–0.036), and equal weights (i.e. each frame was weighted by the same value). The contribution due to vasculature (bv) was subtracted from the ROI TAC prior to the graphical analysis.

#### 2.10.2 Voxelwise Logan’s graphical analysis with arterial ppIF

Parametric images of [^11^C]Ro15-4513 V_T_ were generated from smoothed (isotropic filter with 2.0 mm FWHM) dynamic images and the ppIFs using voxelwise Logan’s graphical analysis with ppIF, as implemented in MICKPM, using the same fixed parameters as listed above.

### 2.11 Model-free analyses, requiring arterial ppIFs

#### 2.11.1 Regional (non-regularised) SA

V_T_s for each ROI were obtained from the dynamic images and the ppIFs using SA (Cunningham et al., 1993, Cunningham and Jones, 1993, Turkheimer et al., 1994), as implemented in MICK using the non-negative least squares (NNLS) algorithm (Lawson and Hanson, 1995). The analysis used a base with 100 logarithmically-spaced functions. The fast frequency boundary was kept at the default value of 0.1 s^-1^. The theoretical slow frequency boundary is based on the decay constant of ^11^C (t_½_≈20 min, decay constant 0005663 s^-1^, log_10_=−3.25). Based on previous work with tracers with relatively slow kinetics (Hammers et al., 2007b, Riaño Barros et al., 2014) and preliminary investigations (Barros et al., 2010b), we changed this to 0.00063 s^-1^ (log_10_=−3.20) in order to reduce noise.

#### 2.11.2 Voxelwise SA

Parametric images of [^11^C]Ro15-4513 V_T_ were generated from the dynamic images and the ppIFs using voxelwise SA as implemented in MICKPM using the NNLS algorithm, with the same number of logarithmic functions and the same fast and slow frequency boundaries as listed above.

### 2.12 Methods not requiring arterial ppIFs

#### 2.12.1 Voxelwise standardised uptake values (SUVs)

Standardised uptake value images (SUVs) were generated from the decay-corrected summation (add) images in SPM8 for frames 16–21 and for frames 22–24, i.e. from 30.5 to 60.5 and from 60.5 to 90.5 min respectively, according to Kenney et al. (1941):

(7) $$\mathrm{SUV}=\frac{\mathrm{radioactivity}\;\times \;\mathrm{weight}}{i\mathrm{nject}\mathrm{ed}\;\mathrm{dose}}$$

#### 2.12.2 Regional SRTM using brainstem or alternatively using cerebellum

GABA_A_ receptors are widespread in the brain, and a true reference region devoid of α5-subunit specific binding does not exist. Attempts have been made to obviate arterial cannulation by using the brain region with the lowest receptor concentration as a pseudo-reference region (Turkheimer et al., 2012). The brainstem and the cerebellum are two of the structures with the lowest concentration of α5 subunits in the human brain (Fritschy and Mohler, 1995, Pirker et al., 2000, Sieghart and Sperk, 2002, Veronese et al., 2016). We therefore used the brainstem and the cerebellum, separately, as a pseudo-reference region in the SRTM (Lammertsma and Hume, 1996) as implemented in MICK, with Nelder-Mead optimisation. We used starting estimates R_I_=0.95, k2a=0.001 min^-1^ and k2a′ (k2RefRegion; efflux rate constant from the reference compartment back to plasma) =0.001 min^-1^. The model reduces to the following equation (Wu and Carson, 2002), from which the binding potential (BP_ND_; (Innis et al., 2007)) can be calculated:

(8) $$C\left(t\right)=R_{1}C_{r}\left(t\right)+R_{1}[k_{2a}^{\prime }-k_{2a}]C_{r}(t)*e^{-k_{2a}t}$$

[where *– convolution operator; C_r_(t) – radioactivity concentration in the reference region tissue; C(t) – total radioactivity concentration timecourse in the tissue; k_2a_ – the apparent k_2_, i.e. k_2_/(1+BP); R_1_ – the relative delivery i.e. the ratio K_1_/K_1_′ where K_1_′ (ml ml^-1^ min^-1^) is the rate constant for the influx of the ligand from the plasma to the reference compartment; t – time (min)].

#### 2.12.3 Voxelwise SRTM2 using brainstem or alternatively using cerebellum

Parametric images of BP_ND_ were generated from the dynamic images using a two-step procedure, SRTM2 (Wu and Carson, 2002) with 100 basis functions (Gunn et al., 1997) in MICKPM. Consistent with the SA, we used beta min=0.00063 s^-1^. We used beta max=0.014 s^-1^ (similar to Gunn et al., 1997). For each participant, k2RefRegion was set to the global median of k2RefRegion estimates derived from a first-pass SRTM, which itself used the same fixed parameters and a tight brain mask (Wu and Carson, 2002).

### 2.13 Global radioactivity concentration

Global radioactivity concentrations were calculated for each decay-corrected, summed radioactivity image (frames 01–24) with an in-house script adapted from SPM (Hammers et al., 2007a), where the global radioactivity concentration is defined as the mean voxel value within a mask. The mask itself is defined as all voxels exceeding one-eighth of the mean value of all voxels in the entire image matrix.

### 2.14 Outcome measures and statistical analyses

#### 2.14.1 Comparison of test and retest injectate data

For the statistical testing we used SPSS for Windows version 22 software (IBM 2013, NY, USA). Injectate data (injected radioactivity, radiochemical purity, co-injected mass of stable ligand, and specific radioactivity at the time of injection) and global radioactivity concentration were compared between test and retest sessions using Student’s paired samples *t*-test (for data with a normal distribution) or the non-parametric Wilcoxon signed-rank test (for data which differed significantly from the normal distribution, i.e. Kolmogorov–Smirnov test p<0.05).

#### 2.14.2 Model fit and within-ROI variability

The median residual sum of squares (RSS) was calculated for each ROI, where available (regional variants), as a summary measure of the fit of the model to the observed data. Alternatively, the median within-subject coefficient of variation (WS-CV) was calculated, where available (voxelwise variants), as a summary measure of the within-ROI variability in the binding parameter.

#### 2.14.3 Reproducibility

To assess test – retest variation (i.e. reproducibility), the median (signed and alternatively absolute) percentage difference between test and retest studies as well as their range was calculated for each ROI, for each variant. The (signed) percentage test-retest differences of binding parameters obtained was calculated according to:

(9) $$\mathrm{Test}-\mathrm{retest}\;\mathrm{difference}=200\;*\;\frac{\left(\mathrm{test}\;\mathrm{value}-\mathrm{retest}\;\mathrm{value}\right)}{\left(\mathrm{test}\;\mathrm{value}+\mathrm{retest}\;\mathrm{value}\right)}$$

Median absolute percentage test – retest differences (MA-TD) of <10% were described as “low”; 10%≤MA-TD<15% were described as “moderate”; 15%≤MA-TD<20% were described as “high”; and MA-TD≥20% were described as “very high”.

#### 2.14.4 Reliability

Reliability was calculated using the intraclass correlation coefficient (ICC; (MacLennan, 1993)):

(10) $$\mathrm{ICC}=\frac{\mathrm{MSBS}-\mathrm{MSWS}}{\mathrm{MSBS}+\left(\mathrm{dfWS}\;\times \;\mathrm{MSWS}\right)}$$

[where MS – mean sum of squares; BS – between-subject; WS – within-subject; and *df* – degrees of freedom]. The ICC is provided to allow assessment of the reliability of the measure as a function of both within-subject variability and between-subject variability; the closer the ICC to 1, the more reliable the variant, i.e. the smaller the within-subject variability of the parameter compared with natural between-subject variability. ICCs were computed in SPSS using the “one-way random” model. We report the “single measures” ICC.

#### 2.14.5 Regional heterogeneity

Finally, the ratio of binding in the highest-binding region (hippocampus) to the lowest-binding non-reference region (occipital lobes) was calculated to allow assessment of each variant’s ability to depict the known heterogeneity in α5 subunit availability across the brain. Ratios (“x”) of 1.5≤x<1.8 were described as “moderate” heterogeneity; 1.8≤x<2.0 were described as “high” heterogeneity; x≥2.0 were described as “very high” heterogeneity.

## 3 Results

### 3.1 Injectate

Details are given in Table 1. There were no significant differences between test and retest studies in terms of the amount of injected radioactivity (median (range): test 440 (430–452) MBq, retest 441 (435–444) MBq, Student’s paired samples *t*-test p=0.90); the radiochemical purity (test 99 (96–99)%, retest 98 (98–99)%, Wilcoxon signed-ranks test p=0.71); the co-injected mass of stable ligand (test 3.0 (2.0–4.4) μg, retest 2.4 (0.4–5.1) μg, Student’s paired samples *t*-test p=0.78); or the specific radioactivity at the time of injection (test 49 (26–72) MBq/ηmol, retest 59 (28–72) MBq/ηmol, Student’s paired samples *t*-test p=0.66).

### 3.2 Global radioactivity concentration

There was no significant difference in global radioactivity concentration between test and retest studies: test 4.65 (range 3.50–5.39) kiloBequerels per millilitre (kBq/ml), retest 4.22 (3.54–4.75) kBq/ml, Student’s paired samples *t*-test p=0.14.

### 3.3 Reproducibility and reliability of blood and PET data quantification

**Fig. 1:**
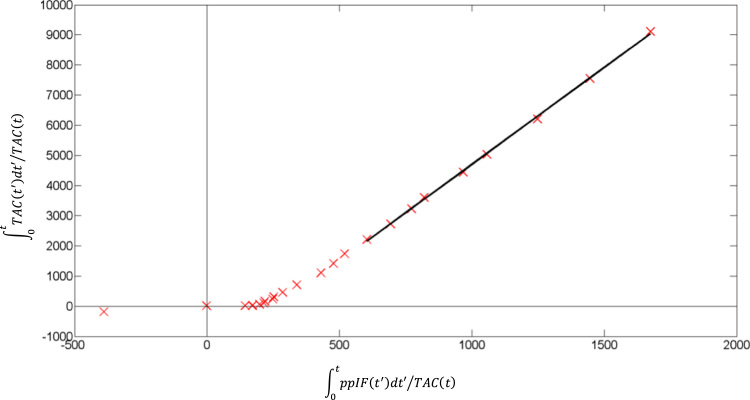
Example of Logan’s graphical analysis plot for a representative participant (2, test scan, for the hippocampus). ppIF(t) – metabolite-corrected plasma radioactivity concentration at time t, TAC(t) – region of interest radioactivity concentration at time t.

**Fig. 2:**
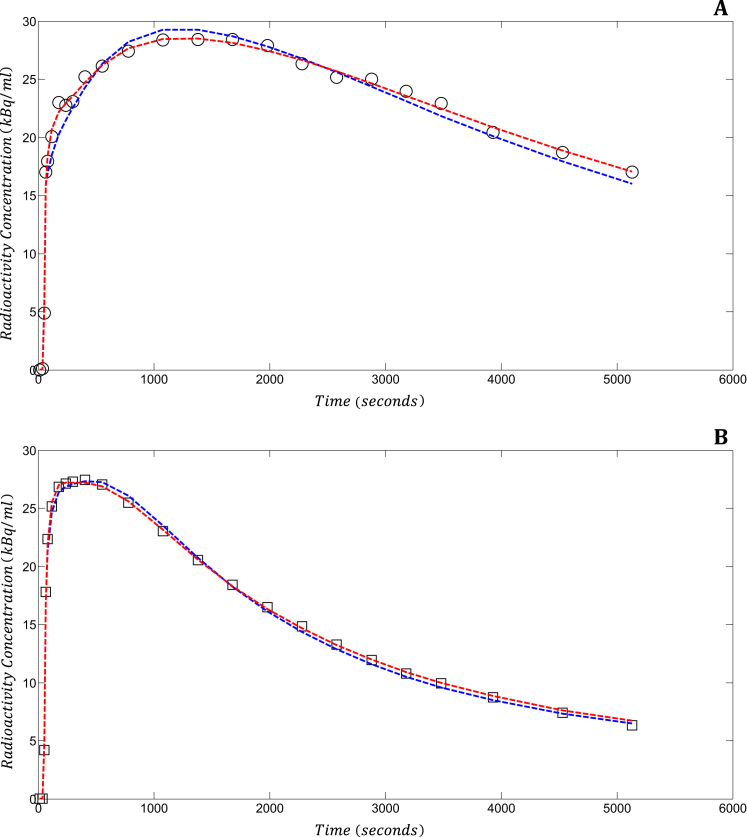
Example of compartmental model fits for a representative participant (1, retest scan) for the hippocampi (A, circles) and the occipital lobes (B, squares). The 2kbv model is depicted by blue dashed lines, and the 4kbv model is depicted by red dashed lines. The 4kbv model fits better than the 2kbv model in the α5-subunit-rich hippocampus. 2kbv – reversible one tissue compartment model with variable blood volume; 4kbv – reversible two tissue compartment model with variable blood volume; kBq/ml – kiloBequerels per millilitre. (For interpretation of the references to color in this figure legend, the reader is referred to the web version of this article).

**Fig. 3:**
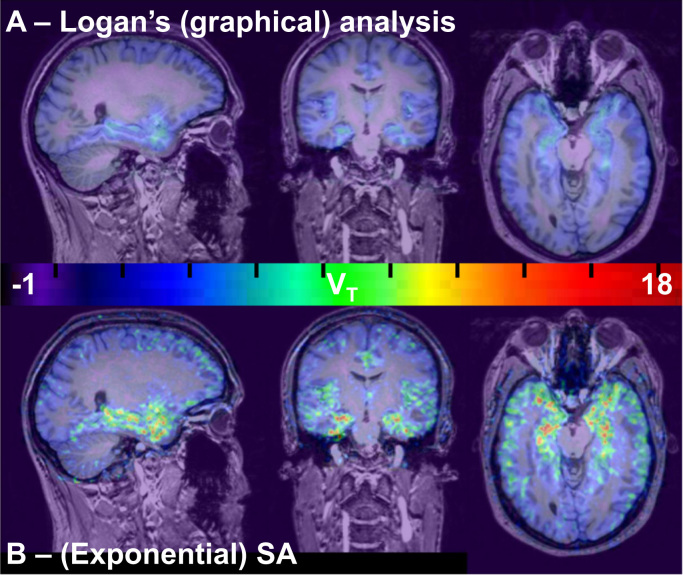
*V*_*T*_ images for participant 2 (test scan), co-registered to the corresponding T1-weighted MRI image. The images were produced by voxelwise Logan’s graphical analysis with ppIF (A – top row), and voxelwise (exponential) SA (B, bottom row). The dynamic data were smoothed (isotropic filter with 2.0 mm FWHM) prior to Logan’s graphical analysis. Note the high binding in temporal regions, and the low binding in the cerebellum. Images are shown in radiological orientation (left on right). The colour bar depicts V_T_. SA – spectral analysis, V_T_ – volume-of-distribution. (For interpretation of the references to color in this figure legend, the reader is referred to the web version of this article).

**Fig. 4:**
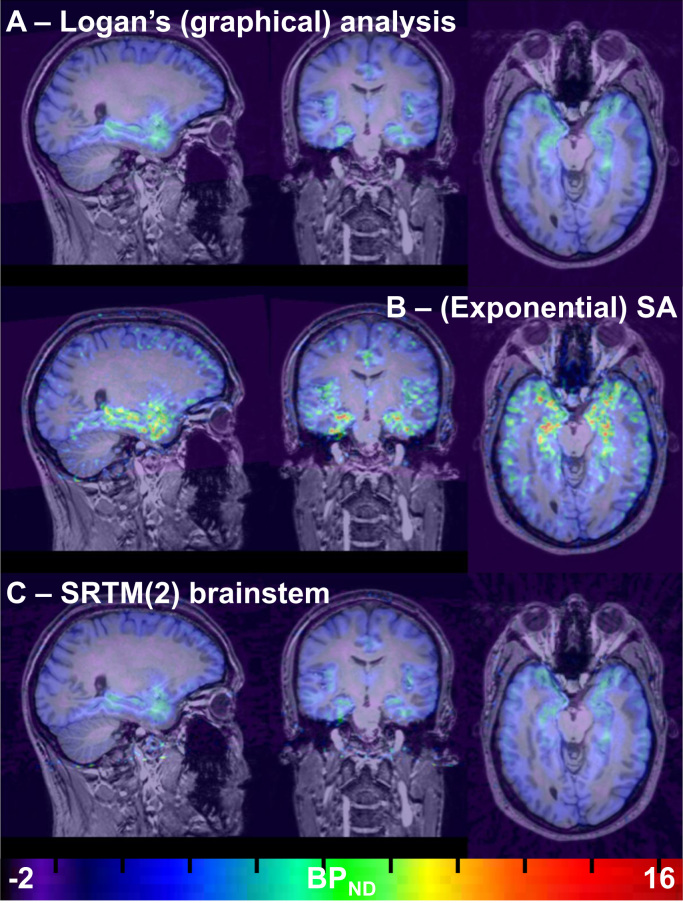
*BP*_*ND*_ images for participant 2 (test scan), co-registered to the corresponding T1-weighted MRI image. For comparison, BP_ND_ images were produced for voxelwise Logan’s graphical analysis with ppIF (A – top row) and voxelwise (exponential) SA (B, second row) by dividing the V_T_ image by the mean of the brainstem (pseudo-reference region) V_T_ and then subtracting 1. BP_ND_ images are shown for SRTM2 using the brainstem (C – third row) as a pseudo-reference region. The dynamic data were smoothed (isotropic filter with 2.0 mm FWHM) prior to Logan’s graphical analysis. Note the high binding in temporal regions, and the low binding in the cerebellum. Images are shown in radiological orientation (left on right). The colour bar depicts BP_ND_. BP_ND_ – binding potential relative to non-displaceable binding, ppIF – (arterial) parent plasma input function; SA – spectral analysis, V_T_ – volume-of-distribution. (For interpretation of the references to color in this figure legend, the reader is referred to the web version of this article).

**Fig. 5:**
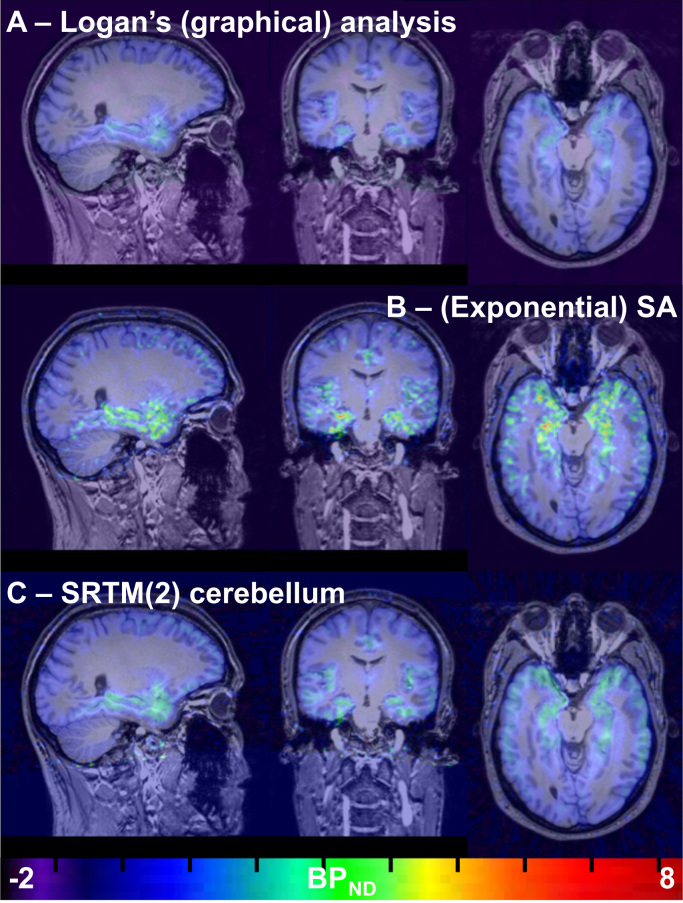
*BP*_*ND*_ images for participant 2 (test scan), co-registered to the corresponding T1-weighted MRI image. For comparison, BP_ND_ images were produced for voxelwise Logan’s graphical analysis with ppIF (A – top row) and voxelwise (exponential) SA (B, second row) by dividing the V_T_ image by the mean of the cerebellum (pseudo-reference region) V_T_ and then subtracting 1. BP_ND_ images are shown for SRTM2 using the cerebellum (C, bottom row) as a pseudo-reference region. The dynamic data were smoothed (isotropic filter with 2.0 mm FWHM) prior to Logan’s graphical analysis. Note the high binding in temporal regions, and the low binding in the cerebellum. Images are shown in radiological orientation (left on right). The colour bar depicts BP_ND_. BP_ND_ – binding potential relative to non-displaceable binding, ppIF – (arterial) parent plasma input function; SA – spectral analysis, V_T_ – volume-of-distribution. (For interpretation of the references to color in this figure legend, the reader is referred to the web version of this article.).

See Sections 2 and 3 of the Supplementary material for details of the parameters derived from the metabolite and plasma-over-blood ratio models, and the six PET quantification methods (12 variants). Fig. 1, Fig. 2, Fig. 3, Fig. 4, Fig. 5 provide examples of the output.

### 3.4 Comparison between analysis variants

The analyses did not produce any outliers for the ROIs (where “outlier” is defined as V_T_ or BP_ND_≤0 and/or WS-CV>50% for regional variants and mean V_T_ or mean BP_ND_≤0 and/or WS-CV>100% for voxelwise variants). For well-performing regional variants, there was no evidence of bias or structure in the weighted residuals.

Table 2 provides an overview of the MA-TDs (%) for the six different methods (12 variants). The MA-TDs were very low to low for all SRTM variants and voxelwise SA (≤5%); MA-TDs were also low (<10%) for most ROIs with the 4kbv model. These variants all had very low test-retest differences in the hippocampus (<5%).

**Table 2:** MA-TDs (median absolute t-rt differences (%)) for participants’ parameter estimates (BP_ND_/SUV/V_T_) obtained with the six different methods (12 variants).

**Method**	**2kbv**	**4kbv**	**Logan’s graphical analysis**	**Logan’s graphical analysis**	**SA**	**SA**	**SUV (30.5–60.5 min)**	**SUV (60.5–90.5 min)**	**SRTM brainstem**	**SRTM2 brainstem**	**SRTM cerebellum**	**SRTM2 cerebellum**
Parameter	V_T_	V_T_	V_T_	V_T_	V_T_	V_T_	SUV	SUV	BP_ND_	BP_ND_	BP_ND_	BP_ND_
Regional/Voxelwise	Regional	Regional	Regional	Voxelwise	Regional	Voxelwise	Voxelwise	Voxelwise	Regional	Voxelwise	Regional	Voxelwise
ACG	28	7	18	19	10	4	11	21	5	6	2	1
Fusiform gyrus	28	14	19	20	14	10	10	15	8	6	5	4
Hippocampus	22	3	13	9	6	2	6	16	3	2	3	2
Inferior frontal gyrus	17	5	8	9	7	5	11	14	5	5	5	4
Insula	27	8	16	17	14	1	12	20	7	6	3	2
Occipital lobes	21	8	17	15	15	8	13	17	4	1	5	3
**Median**	**25**	**8**	**17**	**16**	**12**	**5**	**11**	**17**	**5**	**6**	**4**	**3**
**(iqr)**	**(21–28)**	**(6–8)**	**(14–18)**	**(11–19)**	**(8–14)**	**(3–7)**	**(10–12)**	**(15–19)**	**(4–7)**	**(3–6)**	**(3–5)**	**(2–4)**

2/4kbv=2/4 rate constant compartmental models with variable blood volume, ACG=Anterior cingulate gyrus, BP_ND_=Binding potential relative to non-displaceable binding, iqr=Interquartile range, SUV=Standardised uptake value, ROI=Region-of-interest, SA=Spectral analysis, SRTM=Simplified reference tissue model, *t*-rt=test-retest, V_T_=Volume-of-distribution.

Table 3 provides an overview of the BS-CV (%) for the six different methods (12 variants). The median BS-CVs were moderate for most or all of the ROIs (11–15%) for several variants: 2kbv, 4kbv, voxelwise Logan’s graphical analysis, both regional and voxelwise SA, and SUVs (30.5–60.5 and 60.5–90.5 min). The remaining variants, particularly SRTMs using a pseudo-reference region, were characterised by low (≤10%) BS-CVs for nearly all ROIs.

**Table 3:** Mean between-subject coefficients of variation (BS-CV; %) for participants’ parameter estimates (BP_ND_/SUV/V_T_) obtained with the six different methods (12 variants).

**Method**	**2kbv**	**4kbv**	**Logan’s graphical analysis**	**Logan’s graphical analysis**	**SA**	**SA**	**SUV (30.5–60.5 min)**	**SUV (60.5–90.5 min)**	**SRTM brainstem**	**SRTM2 brainstem**	**SRTM cerebellum**	**SRTM2 cerebellum**
Parameter	V_T_	V_T_	V_T_	V_T_	V_T_	V_T_	SUV	SUV	BP_ND_	BP_ND_	BP_ND_	BP_ND_
Regional/Voxelwise	Regional	Regional	Regional	Voxelwise	Regional	Voxelwise	Voxelwise	Voxelwise	Regional	Voxelwise	Regional	Voxelwise
ACG	15	9	12	12	10	10	16	20	5	5	8	8
Fusiform gyrus	15	26	12	12	14	14	14	19	5	6	7	8
Hippocampus	15	9	10	11	12	11	13	18	8	9	8	11
Inferior frontal gyrus	12	20	10	10	15	12	18	22	8	8	9	10
Insula	14	11	10	10	10	10	14	18	6	6	7	8
Occipital lobes	11	18	9	10	14	10	19	21	7	7	9	9
**Median**	**15**	**15**	**10**	**11**	**13**	**11**	**15**	**20**	**7**	**7**	**8**	**9**
**(iqr)**	**(13–15)**	**(10–20)**	**(10–12)**	**(10–12)**	**(11–14)**	**(10–12)**	**(14–18)**	**(18–21)**	**(5–8)**	**(6–8)**	**(7–9)**	**(8–10)**

2/4kbv=2/4 rate constant compartmental models with variable blood volume, ACG=Anterior cingulate gyrus, BP_ND_=Binding potential relative to non-displaceable binding, iqr=Interquartile range, SUV=Standardised uptake value, ROI=Region-of-interest, SA=Spectral analysis, SRTM=Simplified reference tissue model, *t*-rt=test-retest, V_T_=Volume-of-distribution.

Table 4 provides an overview of the ICCs for the six different methods (12 variants). The median ICC was excellent (>0.80) for both the voxelwise SA, and for the voxelwise SRTM2 with the cerebellum as a pseudo-reference region. Regional SRTM using cerebellum also yielded a good (>0.70) median ICC. Other variants yielded low to moderate (≤0.70) median ICCs. Regarding the hippocampus, the 4kbv model, voxelwise SA, and both regional and voxelwise SRTM/SRTM2 using cerebellum, and voxelwise SRTM2 using the brainstem all yielded excellent (>0.85) median ICCs. Regional SRTM with the brainstem yielded a very good median (0.77) ICC.

**Table 4:** Intraclass Correlation Coefficients (ICCs) for participants’ parameter estimates (BP_ND_/SUV/V_T_) obtained with the six different methods (12 variants).

**Method**	**2kbv**	**4kbv**	**Logan’s graphical analysis**	**Logan’s graphical analysis**	**SA**	**SA**	**SUV (30.5–60.5 min)**	**SUV (60.5–90.5 min)**	**SRTM brainstem**	**SRTM2 brainstem**	**SRTM cerebellum**	**SRTM2 cerebellum**
Parameter	V_T_	V_T_	V_T_	V_T_	V_T_	V_T_	SUV	SUV	BP_ND_	BP_ND_	BP_ND_	BP_ND_
Regional/Voxelwise	Regional	Regional	Regional	Voxelwise	Regional	Voxelwise	Voxelwise	Voxelwise	Regional	Voxelwise	Regional	Voxelwise
ACG	−0.24	0.64	0.08	0.00	0.32	0.91	0.59	0.62	−0.33	−0.18	0.59	0.66
Fusiform gyrus	−0.38	0.45	−0.08	−0.12	0.60	0.71	0.67	0.65	0.22	0.27	0.68	0.64
Hippocampus	−0.52	0.89	−0.07	−0.10	0.35	0.89	0.72	0.72	0.77	0.85	0.95	0.93
Inferior frontal gyrus	−0.41	−0.01	−0.01	−0.02	0.40	0.88	0.76	0.75	0.63	0.66	0.87	0.87
Insula	−0.55	0.51	−0.21	−0.22	−0.73	0.90	0.60	0.63	0.22	0.34	0.74	0.84
Occipital lobes	−0.65	0.36	−0.30	−0.30	−0.18	0.59	0.72	0.68	0.46	0.50	0.63	0.81
**Median**	**−0.47**	**0.48**	**−0.08**	**−0.11**	**0.34**	**0.89**	**0.70**	**0.67**	**0.34**	**0.42**	**0.71**	**0.83**
**(iqr)**	**(−0.54 to −0.39)**	**(−0.38 to 0.61)**	**(−0.18 to −0.03)**	**(−0.20 to −0.04)**	**(−0.06 to 0.39)**	**(0.75–0.90)**	**(0.62–0.72)**	**(0.64–0.71)**	**(0.22–0.59)**	**(0.29–0.62)**	**(0.64–0.84)**	**(0.70–0.86)**
**Hippocampus /Occipital lobes**	1.7	1.9	1.8	1.8	1.8	2.2	1.9	2.5	2.1	1.9	3.2	2.8

2/4kbv=2/4 rate constant compartmental models with variable blood volume, ACG=Anterior cingulate gyrus, BP_ND_=Binding potential relative to non-displaceable binding, iqr=Interquartile range, SUV=Standardised uptake value, ROI=Region-of-interest, SA=Spectral analysis, SRTM=Simplified reference tissue model, *t*-rt=test-retest, V_T_=Volume-of-distribution.

Table 4 also shows the ratio between the hippocampus, which for all variants was the region with highest binding, and a low-binding non-reference region (occipital lobes). Voxelwise SA, regional SRTM using brainstem, and both regional SRTM and voxelwise SRTM2 using the cerebellum all yielded very high ratios (≥2.0).

## 4 Discussion

We describe the test – retest reproducibility and reliability of quantification of the availability of the GABA_A_ receptor α5 subunit in five healthy human participants. Our major finding is that very good to excellent reproducibility of estimates, in terms of percentage test – retest difference, is achievable using regional and voxelwise implementations of the SRTM and also using model-free, voxelwise SA.

Voxelwise SA was the best-performing variant, in terms of ICCs, and one of the best in terms of percentage test – retest difference. This variant also yielded a slightly higher median BS-CV (11%) than SRTM-based variants, and had a high ratio of hippocampal-to-occipital lobe V_T_. We note that voxelwise SA markedly outperformed SA applied to regional TACs. This phenomenon has also been documented for the opioid receptor radioligand [^11^C]diprenorphine (Hammers et al., 2007b) and the cannabinoid receptor type 1 radioligand, [^11^C]MePPEP (Riaño Barros et al., 2014). We suggest that the voxelwise approach benefits from the flexibility to be able to accommodate differences in blood volume, tissue class partial volume and receptor concentration between voxels, in contrast to variants that use the averaged regional TAC.

The assumptions inherent to SA are that: 1) the compartmental systems are strongly connected; 2) the exchange of material with the environment is confined to a single compartment; and 3) there is no possibility for material to pass from one compartment through two or more compartments back to the initial compartment (Schmidt, 1999). There is no evidence to indicate that SA is biased towards or against any particular patient population. An arterial input function is required, as the fit assumes a sum of positive series of convolution integrals of the input function. One advantage of SA is that it is “data driven”, i.e. *a priori* model selection is not required. Like all voxel-based methods, the generation of parametric V_T_ images via voxelwise SA has the added advantage of allowing whole-brain surveys in diseases where the exact localisation of pathology is not known, e.g. refractory focal epilepsy.

Of the compartmental models, the 2kbv model had a very high median percentage test – retest variability (MA-TD; 25%); whilst the 4kbv model had an acceptable MA-TD (8%). However, a wide range of percentage test – retest variability was observed across participants for each region, other than in the hippocampus (3%, range −2–7%). These data are in keeping with previous findings, in which the fits with two-tissue compartment models were better than those seen with one-tissue compartment models (Asai et al., 2009, Myers et al., 2012, Myers et al., 2016).

While [^11^C]Ro15-4513 has highest affinity for GABA_A_ receptors containing α5 subunits (Lingford-Hughes et al., 2002), it also binds to GABA_A_ receptors containing α1 and other α subunits, albeit with approximately 10–15 times lower affinity (Hadingham et al., 1993, Luddens et al., 1994, Myers et al., 2012, Stokes et al., 2013). Recently, human heterologous competition data acquired from healthy males using the α5-subunit-selective negative allosteric modulator, Basmisanil (RG1662), suggested that α5-specific binding accounts for 60–70% of the specific binding in most regions (Myers et al., 2016). As the regional distribution of α subunits overlaps, the tissue kinetics, model fits and hence reliability will vary according to the proportion of subunits (Maeda et al., 2003, Myers et al., 2012). Model-free quantification, such as with SA, offers flexibility to deal with the complex compartmentalisation of the radioligand targets (Myers et al., 2012, Riaño Barros et al., 2014).

Logan’s graphical analyses had a very high MA-TD, whether applied to regional TACs or on a voxel-by-voxel basis. It is possible that more than nine frames are required to accurately fit the plot, although we did smooth the dynamic images before voxelwise analyses. Also, the analyses assumed a fixed blood volume contribution of 0.028, the median derived from multiple regions and scans, which cannot be correct for each ROI in each participant.

In the present study, we quantified the total V_T_, rather than attempting to isolate the presumed α5-subunit-specific volume-of-distribution (V_s_), for example via bandpass SA (Stokes et al., 2014). However, accurate isolation of the V_s_ is challenging and is vulnerable to the effects of tissue heterogeneity and noise. Whilst V_s_ appears to exhibit a tight relationship with the ‘true’ α5-subunit-specific V_T_ in regions with moderate or high α5 subunit concentration, the total V_T_ also exhibits a tight, linear association (Myers et al., 2016).

As expected, our analyses revealed that the reproducibility of [^11^C]Ro15-4513 V_T_ was sensitive to multiple methodological choices, e.g. derivation of the input function and method used to calculate the weighting factors (e.g. Yaqub et al., 2006; data not shown).

Non-invasive PET studies are preferable in both research and clinical studies, in order to avoid the discomfort and slight risks attributable to arterial cannulation, a procedure which demands expertise. GABA_A_ receptor α5 subunits are expressed throughout the brain, and a true reference region does not exist. Here we used the brainstem and alternatively the cerebellum as pseudo-reference regions, based on their near-negligible expression of the α5 subunit (Fritschy and Mohler, 1995, Pirker et al., 2000, Sieghart and Sperk, 2002, Veronese et al., 2016). This approach is supported by recent data that suggest the V_s_ is low in the cerebellum and extremely low in the pons (Myers et al., 2016). In the present study, both variants yielded reproducible data, in terms of percentage test – retest difference, whether applied to regional TACs or on a voxel-by voxel basis. BS-CV was low, however, which perhaps reflects a bias of the reference region methods. The actual BP_ND_ values were much lower for the SRTMs when using cerebellum as the pseudo-reference rather than the brainstem, which probably reflects greater α5-subunit-specific binding in the former (Myers et al., 2016). A wider range of percentage test – retest difference was seen for the brainstem than for the cerebellum with compartmental models. We observed lower signal-to-noise ratio, i.e. noisier time-activity curves, for the smaller brainstem ROI, which impaired model fitting.

Whilst the recent Basmisanil (RG1662) blocking study found a tight, linear relationship between BP_ND_ and the ‘true’ α5-subunit-specific volume-of-distribution with both variants (Myers et al., 2016), SRTMs should only be used if even very small intra- or between-subject variations in the amount of GABA_A_ receptor-specific binding in these pseudo-reference regions can be excluded. It might be possible to improve on these results by utilising a sub-region of the brainstem or cerebellum, or via a more sophisticated pseudo-reference region approach (e.g. Turkheimer et al., 2012).

SUVs can constitute a simple and reliable measure of radioligand binding that obviates the need for arterial blood sampling (Riaño Barros et al., 2014). In the present study, [^11^C]Ro15-4513 SUVs were moderately reproducible overall (median MA-TD 11%), but had a wide range in percentage test – retest difference for most regions. The SUVs were moderately reliable (median ICC 0.70), which is partly attributable to the large BS-CV (median 15%). Overall these data suggest that factors other than weight and injected dose significantly influence reproducibility. Given the performance of voxelwise SA with arterial input function, and the moderately-high MA-TD we observed in the area under the metabolite model curve (12%, see Supplementary material, Table 2), we hypothesise that such factors include the rate of metabolism of the parent radioligand.

The present study is limited by the sample size; in particular ICCs must be treated with caution when using paired data acquired from eight or less participants (Walter et al., 1998, Shoukri et al., 2004). As the free fraction of parent radioligand in the plasma was not quantified, we cannot comment on the reproducibility or reliability of BP_f_ or VT_f_ (Innis et al., 2007). However, voxelwise SA based on arterial ppIFs that were not corrected for plasma free fraction still yielded reproducible and reliable V_T_ data. The test – retest scan interval was short, but varied from a week to two months between participants; this variable was not associated with reproducibility.

The lack of females in our study population is an additional limitation. To the best of our knowledge, the potential influence of the menstrual cycle on the availability of the GABA_A_ receptor or of the α5 subunit in particular has not been studied. However, the menstrual cycle influences GABA concentration in the frontal lobe, as measured by proton magnetic resonance spectroscopy (e.g. Harada et al., 2011; De Bondt et al., 2015), which could conceivably lower test – retest reproducibility. Hence, confirmation of our findings in women would be desirable.

## 5 Conclusions

Quantification of [^11^C]Ro15-4513 binding shows very good to excellent reproducibility with SRTMs and voxelwise SA. Quantification of binding in the α5-subunit-rich hippocampus is particularly reliable. Whilst SA necessitates arterial blood sampling, it is preferable to the SRTMs due to the lack of a true reference region. [^11^C]Ro15-4513 PET is well-placed as a tool to study the availability of the GABA_A_ receptor α5 subunit in health and neuropsychiatric disease.

## Conflicts of interest

The authors do not report any conflicts of interest.
