# Supplementary material for: Test-retest reproducibility of quantitative binding measures of [11C]Ro15-4513, a PET ligand for GABAA receptors containing alpha5 subunits
Source: Neuroimage. 2017 May 15;152:270–82. doi: 10.1016/j.neuroimage.2016.12.038 (PMC5440177; doi:10.1016/j.neuroimage.2016.12.038)
Supplement: Supplementary file 1 — Supplementary material [file mmc1.docx]

Test-retest reproducibility of quantitative binding measures of [^11^C]Ro15 4513, a PET ligand for GABA_A_ receptors containing alpha 5 subunits:

Supplementary Material

# 1. Comparison of Clickfit versus MICK

MICK (Modelling, Input functions and Compartmental Kinetics) version 5.2 software (available on request from Rainer Hinz, Wolfson Molecular Imaging Centre, University of Manchester, Manchester, UK) uses scripts derived from Clickfit, which was widely used at the Medical Research Council Clinical Sciences Centre (Hammersmith Hospital, London, UK; e.g. ([Hammers et al., 2007](#_ENREF_1); [Riaño Barros et al., 2014](#_ENREF_2))). Comparison of volume-of distribution (V_T_) and binding potential (relative to non-displaceable binding; BP_ND_) estimates derived from Clickfit and MICK was made for four participants across five regions-of-interest (ROIs), using: 1) reversible compartmental models with one and two tissue compartments and variable blood volume (2kbv, 4kbv); 2) regional exponential spectral analysis (SA); and 3) the regional simplified reference tissue model (SRTM) using cerebellum as a pseudo-reference region.

Excellent correspondence between estimates was observed for all methods (example provided in Table 2). Across ROIs, the Pearson’s correlation coefficient squared (R^2^) ranged from 0.91 (regional SA) to > 0.99 (2kbv). Two instances were noted in which a binding parameter estimate derived from MICK (one with 4kbv, one with regional SA) appeared less aberrant than the corresponding estimate derived from Clickfit. Exclusion of these two datapoints increased the R^2^ to > 0.99 for both methods.

|  | **Test scan** | | | **Retest scan** | | |
| --- | --- | --- | --- | --- | --- | --- |
|  | **Clickfit V_T_** | **MICK**  **V_T_** | **Absolute Percentage Difference in V_T_** | **Clickfit V_T_** | **MICK**  **V_T_** | **Absolute Percentage Difference in V_T_** |
| **Participant 1** | 7.91 | 8.06 | 1.96 | 7.78 | 7.78 | 0.01 |
| **Participant 2** | 6.47 | 6.41 | 0.95 | 5.80 | 5.80 | 0.00 |
| **Participant 3** | 7.65 | 7.65 | 0.03 | 7.15 | 7.14 | 0.04 |
| **Participant 4** | 8.00 | 8.01 | 0.08 | 7.74 | 7.74 | 0.03 |

*Table 1 Example of hippocampal V_T_ estimates derived from the 4kbv compartmental model, as implemented in Clickfit and MICK.* Clickfit data was not available for Participant 5. The absolute percentage differences in V_T_ were calculated as: 200 * ((test – retest value)/(test + retest value)). MICK – Modelling, Input functions and Compartmental Kinetics version 5.2 software, V_T_ – volume-of-distribution.

Here, the default MICK parameters were used (maximum of 3000 function evaluations, maximum of 3000 iterations, tolerance 1 x 10^-3^, and fraction 1 x 10^-7^). It is possible that variation/refinement of these parameter settings might yield even greater correspondence with Clickfit.

# 2. Reproducibility and reliability of blood data quantification

The area under the metabolite model curve (AUC_metab_) had a moderately-high median absolute test – retest percentage difference (MA-TD; 12%). In contrast, the area under the plasma-over-blood ratio model curve (AUC_pob_) yielded extremely consistent data (MA-TD 0%). The intraclass correlation coefficients (ICCs) were -0.09 and 0.92, respectively.

|  | **Median**  **AUC** | **iqr** | **Min** | **Max** | **Median RSS** | **Median signed**  **% t-rt diff** | **% t-rt diff range** | **MA-TD (%)** | **Mean %**  **BS-CV** | **ICC** |
| --- | --- | --- | --- | --- | --- | --- | --- | --- | --- | --- |
| **AUC_metab_** | 1468.50 | 1289.08 – 1578.40 | 1160.90 | 1891.90 | 3.63 x 10^-3^ | 9 | -12 – 35 | 12 | 15 | -0.09 |
| **AUC_pob_** | 7732.15 | 7330.20 – 7771.53 | 7236.20 | 7976.90 | 2.7 x 10^-3^ | 0 | -1 – 3 | 0 | 4 | 0.92 |

*Table 2 Participants’ metabolite model and plasma-over-blood ratio models.* AUC_metab_ – area under the metabolite model curve; AUC_pob_ – area under the plasma-over-blood ratio model curve; BS-CV – between-subject coefficient of variation, diff – difference, ICC – intraclass correlation coefficient, iqr – interquartile range, MA-TD – median absolute test – retest difference, Max – maximum, Min – minimum, RSS – residual sum of squares; t-rt – test – retest.

# 3. Reproducibility and reliability of PET data quantification

The following sections describe the binding parameter estimates derived from the six quantification methods (12 variants). A synthetic overview and comparison of the variants is provided in the manuscript proper, Section “3.4 Comparison between analysis variants”.

## 3.1 Reversible compartmental models, requiring arterial parent plasma input functions (ppIFs)

*3.1.1 2kbv*

An example of the model fit is provided in the manuscript proper, Figure 2. The method yielded inconsistent data for all ROIs, with very high MA-TDs for several ROIs (> 20%; Table 3). ICCs ranged between -0.65 and -0.24, with a median (iqr) of -0.47 (-0.54 – -0.39).

|  | **Median**  **V_T_** | **iqr** | **Min** | **Max** | **Median RSS** | **Median signed**  **% t-rt diff** | **% t-rt diff range** | **MA-TD**  **(%)** | **Mean %**  **BS-CV** | **ICC** |
| --- | --- | --- | --- | --- | --- | --- | --- | --- | --- | --- |
| **ACG** | 6.06 | 5.27 – 6.66 | 4.80 | 6.96 | 0.52 | 3 | -29 – 28 | 28 | 15 | -0.24 |
| **Fusiform gyrus** | 5.77 | 4.87 – 6.36 | 4.62 | 6.79 | 0.55 | 1 | -32 – 28 | 28 | 15 | -0.38 |
| **Hippocampus** | 6.20 | 5.44 – 7.14 | 5.15 | 7.48 | 1.53 | -4 | -37 – 22 | 22 | 15 | -0.52 |
| **Inferior frontal gyrus** | 4.59 | 4.17 – 5.00 | 4.02 | 5.56 | 0.39 | 1 | -31 – 17 | 17 | 12 | -0.41 |
| **Insula** | 5.84 | 5.10 – 6.55 | 4.94 | 6.79 | 0.70 | -1 | -30 - 27 | 27 | 14 | -0.55 |
| **Occipital lobes** | 3.63 | 3.31 – 3.83 | 3.13 | 4.28 | 0.38 | 0 | -29 – 21 | 21 | 11 | -0.65 |
| Median (iqr): |  |  |  |  | **0.54**  **(0.42 – 0.66)** |  |  | **25**  **(21 – 28)** | **15**  **(13 – 15)** | **-0.47**  **(-0.54 – -0.39)** |
|  |  |  |  |  |  |  |  |  |  |  |
| **Brainstem** | 1.01 | 0.96 – 1.04 | 0.86 | 1.18 | 0.14 | 2 | -31 - 13 | 13 | 9 | -0.74 |
| **Cerebellum** | 2.18 | 2.07 – 2.25 | 2.00 | 2.68 | 0.20 | -3 | -26 – 11 | 15 | 8 | -0.41 |

*Table 3 Participants’ V_T_ (2kbv compartmental model).* 2kbv – reversible compartmental model with two compartments (one tissue compartment) and variable blood volume, ACG – anterior cingulate gyrus, BS-CV – between-subject coefficient of variation, diff – difference, ICC – intraclass correlation coefficient, iqr – interquartile range, MA-TD – median absolute test – retest difference, Max – maximum, Min – minimum, RSS – residual sum of squares; t-rt – test – retest, V_T_ – volume-of-distribution.

*3.1.2 4kbv*

An example of the model fit is provided in the manuscript proper, Figure 2. The 4kbv method yielded inconsistent data for several ROIs, with very wide range of signed percentage test – retest (t-rt) differences (Table 4, “% t-rt diff range” column). ICCs ranged between -0.01 and 0.89, with a median (iqr) of 0.48 (0.38 – 0.61). Quantification in the hippocampus was very reproducible and very reliable (MA-TD 3%, ICC 0.89).

|  | **Median**  **V_T_** | **iqr** | **Min** | **Max** | **Median RSS** | **Median signed**  **% t-rt diff** | **% t-rt diff range** | **MA-TD**  **(%)** | **Mean %**  **BS-CV** | **ICC** |
| --- | --- | --- | --- | --- | --- | --- | --- | --- | --- | --- |
| **ACG** | 6.74 | 6.18 – 7.08 | 5.64 | 7.37 | 0.13 | 5 | -9 – 14 | 7 | 9 | 0.64 |
| **Fusiform gyrus** | 6.30 | 5.74 – 6.58 | 1.65 | 7.27 | 0.18 | -13 | -103 – 14 | 14 | 26 | 0.45 |
| **Hippocampus** | 7.47 | 7.15 – 7.77 | 5.98 | 8.01 | 0.17 | 3 | -2 – 7 | 3 | 9 | 0.89 |
| **Inferior frontal gyrus** | 5.14 | 4.55 – 5.47 | 2.46 | 5.72 | 0.07 | 1 | -77 – 7 | 5 | 20 | -0.01 |
| **Insula** | 6.74 | 6.18 – 6.90 | 5.42 | 8.03 | 0.09 | 8 | -6 - 17 | 8 | 11 | 0.51 |
| **Occipital lobes** | 3.97 | 3.74 – 4.27 | 3.21 | 5.92 | 0.08 | -1 | -30 – 29 | 8 | 18 | 0.36 |
| Median (iqr): |  |  |  |  | **0.11**  **(0.08 – 0.16)** |  |  | **8**  **(6 – 8)** | **15**  **(10 – 20)** | **0.48**  **(-0.38 – 0.61)** |
|  |  |  |  |  |  |  |  |  |  |  |
| **Brainstem** | 1.0 | 0.8 – 1.12 | 0.84 | 1.28 | 0.07 | -3 | -42 – 23 | 23 | 15 | -0.50 |
| **Cerebellum** | 2.17 | 1.99 – 2.38 | 1.95 | 3.12 | 0.07 | -3 | -42 – 15 | 15 | 14 | -0.24 |

*Table 4 Participants’ V_T_ (4kbv compartmental model).* 2kbv – reversible compartmental model with three compartments (two tissue compartments) and variable blood volume, ACG – anterior cingulate gyrus, diff – difference, BS-CV – between-subject coefficient of variation, ICC – intraclass correlation coefficient, iqr – interquartile range, MA-TD – median absolute test – retest difference, Max – maximum, Min – minimum, RSS – residual sum of squares; t-rt – test – retest, V_T_ – volume-of-distribution.

**3.2 Graphical analyses, requiring arterial ppIFs**

*3.2.1 Regional Logan’s graphical analysis with arterial ppIF*

An example of the Logan’s analysis graphical plot is provided in the manuscript proper, Figure 1. The MA-TD was 8% for the inferior frontal gyrus (Table 5, “MA-TD” column). However, the variant yielded inconsistent data for the other non-reference ROIs, with moderate to high MA-TDs (≥ 13%; Table 5). ICCs ranged between -0.30 and 0.08, with a median (iqr) of -0.08 (-0.18 – -0.03).

|  | **Median**  **V_T_** | **iqr** | **Min** | **Max** | **Median RSS** | **Median signed**  **% t-rt diff** | **% t-rt diff range** | **MA-TD (%)** | **Mean %**  **BS-CV** | **ICC** |
| --- | --- | --- | --- | --- | --- | --- | --- | --- | --- | --- |
| **ACG** | 6.35 | 5.73 – 7.11 | 5.48 | 7.30 | 4859 | -1 | -23 – 18 | 18 | 12 | 0.08 |
| **Fusiform gyrus** | 6.13 | 5.54 – 6.59 | 5.04 | 7.19 | 15719 | -2 | -24 – 19 | 19 | 12 | -0.08 |
| **Hippocampus** | 6.90 | 6.49 – 7.60 | 5.88 | 8.04 | 15798 | -6 | -23 – 13 | 13 | 10 | -0.07 |
| **Inferior frontal gyrus** | 4.90 | 4.56 – 5.24 | 4.14 | 5.72 | 3536 | -2 | -23 – 8 | 8 | 10 | -0.01 |
| **Insula** | 6.24 | 5.86 – 6.89 | 5.43 | 7.12 | 5112 | -4 | -21 – 16 | 16 | 10 | -0.21 |
| **Occipital lobes** | 3.81 | 3.57 – 4.02 | 3.34 | 4.46 | 4792 | -4 | -22 – 17 | 17 | 9 | -0.30 |
| Median (iqr): |  |  |  |  | **4986**  **(4809 –13067)** |  |  | **17**  **(14 -18)** | **10**  **(10 – 12)** | **-0.08**  **(-0.18 – -0.03)** |
|  |  |  |  |  |  |  |  |  |  |  |
| **Brainstem** | 1.11 | 0.97 – 1.19 | 0.93 | 1.38 | 19770 | -4 | -36 – 13 | 13 | 12 | -0.48 |
| **Cerebellum** | 2.27 | 2.09 – 2.43 | 2.04 | 2.73 | 5543 | -6 | -22 – 12 | 12 | 9 | -0.16 |

*Table 5 Participants’ V_T_ (regional Logan’s graphical analysis with arterial ppIF).* ACG – anterior cingulate gyrus, diff – difference, BS-CV – between-subject coefficient of variation, ICC – intraclass correlation coefficient, iqr – interquartile range, MA-TD – median absolute test – retest difference, Max – maximum, Min – minimum, RSS – residual sum of squares; t-rt – test – retest, V_T_ – volume-of-distribution.

*3.2.2 Voxelwise Logan’s graphical analysis with arterial ppIF*

This variant yields parametric images of V_T_; an example is provided in manuscript proper, Figure 3. The variant yielded inconsistent data for most of the examined non-reference ROIs, with moderate to high MA-TDs (≥ 15%) for all but the hippocampus and inferior frontal gyrus (Table 6). ICCs ranged between -0.30 and 0.00, with a median (iqr) of -0.11 (-0.20 – -0.04).

|  | **Median**  **V_T_** | **iqr** | **Min** | **Max** | **Median %**  **WS-CV** | **Median signed**  **% t-rt diff** | **% t-rt diff range** | **MA-TD**  **(%)** | **Mean %**  **BS-CV** | **ICC** |
| --- | --- | --- | --- | --- | --- | --- | --- | --- | --- | --- |
| **ACG** | 6.23 | 5.57 – 6.95 | 5.30 | 7.05 | 21 | -1 | -23 – 20 | 19 | 12 | 0.00 |
| **Fusiform gyrus** | 5.82 | 5.10 – 6.30 | 4.81 | 6.39 | 28 | -2 | -24 – 20 | 20 | 12 | -0.12 |
| **Hippocampus** | 6.64 | 6.06 – 7.16 | 5.51 | 7.77 | 18 | -5 | -25 – 9 | 9 | 11 | -0.10 |
| **Inferior frontal gyrus** | 4.85 | 4.48 – 5.18 | 4.06 | 5.65 | 19 | -2 | -23 – 9 | 9 | 10 | -0.02 |
| **Insula** | 6.11 | 5.65 – 6.76 | 5.30 | 6.97 | 19 | -4 | -21 – 17 | 17 | 10 | -0.22 |
| **Occipital lobes** | 3.76 | 3.47 – 4.00 | 3.29 | 4.40 | 19 | -2 | -22 – 15 | 15 | 10 | -0.30 |
| Median (iqr): |  |  |  |  | **19**  **(19 – 21)** |  |  | **16**  **(11 – 19)** | **11**  **(10 – 12)** | **-0.11**  **(-0.20 – -0.04)** |
|  |  |  |  |  |  |  |  |  |  |  |
| **Brainstem** | 1.11 | 0.98 – 1.18 | 0.37 | 1.28 | 41 | -2 | -108 – 15 | 15 | 24 | -0.36 |
| **Cerebellum** | 2.22 | 2.03 – 2.36 | 1.94 | 2.60 | 24 | -5 | -24 – 12 | 12 | 9 | -0.43 |

*Table 6 Participants’ V_T_ (voxelwise Logan’s graphical analysis with arterial ppIF).* ACG – anterior cingulate gyrus, diff – difference, BS-CV – between-subject coefficient of variation, ICC – intraclass correlation coefficient, iqr – interquartile range, MA-TD – median absolute test – retest difference, Max – maximum, Min – minimum, t-rt – test – retest, V_T_ – volume-of-distribution; WS-CV – within-subject coefficient of variation.

**3.3 Model-free analyses, requiring arterial ppIFs**

*3.3.1 Regional (non-regularised) SA*

The variant yielded inconsistent data for several ROIs, with moderate to high MA-TDs (≥ 10%; Table 7). ICCs ranged between -0.73 and 0.60, with a median (iqr) of 0.34 (-0.06 – 0.39). The MA-TD for the hippocampus and the inferior frontal gyrus were low (6%, 7%), however the range of signed differences was wide (e.g. -3 – 27%, “% t-rt diff range” column).

|  | **Median**  **V_T_** | **iqr** | **Min** | **Max** | **Median RSS** | **Median signed**  **% t-rt diff** | **% t-rt diff range** | **MA-TD**  **(%)** | **Mean %**  **BS-CV** | **ICC** |
| --- | --- | --- | --- | --- | --- | --- | --- | --- | --- | --- |
| **ACG** | 6.61 | 6.28 – 6.91 | 5.69 | 7.94 | 0.10 | 6 | -18 - 14 | 10 | 10 | 0.32 |
| **Fusiform gyrus** | 6.30 | 5.75 – 5.68 | 4.42 | 7.27 | 0.18 | -13 | -16 – 14 | 14 | 14 | 0.60 |
| **Hippocampus** | 7.19 | 6.49 – 7.86 | 5.49 | 8.14 | 0.13 | 6 | -3 - 27 | 6 | 12 | 0.35 |
| **Inferior frontal gyrus** | 5.16 | 4.59 – 5.62 | 3.67 | 5.92 | 0.05 | -1 | -37 - 7 | 7 | 15 | 0.40 |
| **Insula** | 6.77 | 6.07 – 6.92 | 5.91 | 8.08 | 0.05 | -2 | -16 – 31 | 14 | 10 | -0.73 |
| **Occipital lobes** | 3.97 | 3.39 – 4.09 | 2.69 | 4.59 | 0.04 | -15 | -42 – 14 | 15 | 14 | -0.18 |
| Median (iqr): |  |  |  |  | **0.08**  **(0.05 – 0.12)** |  |  | **12**  **(8 – 14)** | **13**  **(11 – 14)** | **0.34**  **(-0.06 – 0.39)** |
|  |  |  |  |  |  |  |  |  |  |  |
| **Brainstem** | 1.07 | 0.98 – 1.16 | 0.83 | 1.31 | 0.03 | -6 | -31 - 23 | 17 | 15 | 0.17 |
| **Cerebellum** | 2.20 | 2.07 – 2.40 | 1.95 | 2.69 | 0.03 | 5 | -31 - 17 | 17 | 11 | -0.63 |

*Table 7 Participants’ V_T_ (regional “classic” (non-regularised) SA).* ACG – anterior cingulate gyrus, diff – difference, BS-CV – between-subject coefficient of variation, ICC – intraclass correlation coefficient, iqr – interquartile range, MA-TD – median absolute test – retest difference, Max – maximum, Min – minimum, RSS – residual sum of squares; SA – (exponential) spectral analysis; t-rt – test – retest, V_T_ – volume-of-distribution.

*3.3.2 Voxelwise SA*

This variant yields parametric images of V_T_; an example is provided in manuscript proper, Figure 3. The variant yielded consistent data for most ROIs, with low MA-TDs (< 10%; Table 8). ICCs ranged between 0.59 and 0.91, with a median (iqr) of 0.89 (0.75 – 0.90). Quantification in the hippocampus was very reproducible and very reliable (MA-TD 2%, ICC 0.89).

|  | **Median**  **V_T_** | **iqr** | **Min** | **Max** | **Median %**  **WS-CV** | **Median signed**  **% t-rt diff** | **% t-rt diff range** | **MA-TD (%)** | **Mean %**  **BS-CV** | **ICC** |
| --- | --- | --- | --- | --- | --- | --- | --- | --- | --- | --- |
| **ACG** | 7.75 | 7.22 – 8.31 | 6.51 | 8.77 | 32 | -1 | -7 – 6 | 4 | 10 | 0.91 |
| **Fusiform gyrus** | 7.69 | 7.18 – 8.33 | 6.08 | 9.64 | 40 | -6 | -10 – 16 | 10 | 14 | 0.71 |
| **Hippocampus** | 9.56 | 8.75 – 10.11 | 7.92 | 10.49 | 38 | 0 | -11 – 3 | 2 | 11 | 0.89 |
| **Inferior frontal gyrus** | 5.83 | 5.57 – 6.36 | 4.58 | 6.51 | 27 | -3 | -9 – 5 | 5 | 12 | 0.88 |
| **Insula** | 7.95 | 7.56 – 8.36 | 6.46 | 8.62 | 31 | 0 | -8 – 7 | 1 | 10 | 0.90 |
| **Occipital lobes** | 4.43 | 4.18 – 4.86 | 3.74 | 5.15 | 27 | -6 | -14 – 10 | 8 | 10 | 0.59 |
| Median (iqr): |  |  |  |  | **32**  **(28 – 37)** |  |  | **5**  **(3 – 7)** | **11**  **(10 – 12)** | **0.89 (0.75 – 0.90)** |
|  |  |  |  |  |  |  |  |  |  |  |
| **Brainstem** | 1.45 | 1.40 – 1.53 | 0.99 | 2.01 | 76 | 5 | -30 – 36 | 24 | 18 | -0.89 |
| **Cerebellum** | 2.69 | 2.45 – 3.05 | 2.22 | 3.54 | 41 | 8 | -22 – 21 | 20 | 15 | 0.32 |

*Table 8 Participants’ V_T_ (voxelwise “classic” SA).* ACG – anterior cingulate gyrus, diff – difference, BS-CV – between-subject coefficient of variation, ICC – intraclass correlation coefficient, iqr – interquartile range, MA-TD – median absolute test – retest difference, Max – maximum, Min – minimum, SA – (exponential) spectral analysis; t-rt – test – retest, V_T_ – volume-of-distribution; WS-CV – within-subject coefficient of variation.

**3.4 Methods not requiring arterial ppIFs**

*3.4.1 Voxelwise standardised uptake values (SUVs; 30.5 – 60.5 minutes)*

The variant yielded moderate MA-TDs for most non-reference ROIs (10 – 13 %; Table 9). ICCs ranged from 0.59 to 0.76, with a median (iqr) of 0.70 (0.62 – 0.72). The MA-TD in the hippocampus was low (6%), however the range of signed percentage t-rt differences was quite wide (-1 – 18%, “% t-rt diff range” column).

|  | **Median**  **SUV** | **iqr** | **Min** | **Max** | **Median %**  **WS-CV** | **Median signed**  **% t-rt diff** | **% t-rt diff range** | **MA-TD (%)** | **Mean %**  **BS-CV** | **ICC** |
| --- | --- | --- | --- | --- | --- | --- | --- | --- | --- | --- |
| **ACG** | 3.58 | 3.30 – 4.24 | 2.72 | 4.43 | 22 | 11 | -4 – 24 | 11 | 16 | 0.59 |
| **Fusiform gyrus** | 3.50 | 3.05 – 3.99 | 2.77 | 4.08 | 29 | 10 | -1 – 19 | 10 | 14 | 0.67 |
| **Hippocampus** | 3.93 | 3.41 – 4.35 | 3.23 | 4.48 | 18 | 6 | -1 – 18 | 6 | 13 | 0.72 |
| **Inferior frontal gyrus** | 2.72 | 2.28 – 3.21 | 2.14 | 3.37 | 21 | 11 | -6 – 24 | 11 | 18 | 0.76 |
| **Insula** | 3.66 | 3.19 – 4.07 | 2.89 | 4.27 | 20 | 12 | -3 – 20 | 12 | 14 | 0.60 |
| **Occipital lobes** | 1.94 | 1.69 – 2.31 | 1.50 | 2.51 | 23 | 13 | -6 – 22 | 13 | 19 | 0.72 |
| Median (iqr): |  |  |  |  | **22**  **(20 – 23)** |  |  | **11**  **(10 – 12)** | **15**  **(14 –18)** | **0.70 (0.62 – 0.72)** |
|  |  |  |  |  |  |  |  |  |  |  |
| **Brainstem** | 0.49 | 0.46 – 0.55 | 0.41 | 0.65 | 87 | 10 | -7 – 18 | 10 | 15 | 0.71 |
| **Cerebellum** | 1.03 | 0.91 – 1.19 | 0.81 | 1.44 | 48 | 16 | -9 – 21 | 16 | 20 | 0.72 |

*Table 9 Participants’ standardised uptake values (SUVs; voxelwise 30.5 – 60.5 minutes).*  ACG – anterior cingulate gyrus, diff – difference, BS-CV – between-subject coefficient of variation, ICC – intraclass correlation coefficient, iqr – interquartile range, MA-TD – median absolute test – retest difference, Max – maximum, Min – minimum, t-rt – test – retest, WS-CV – within-subject coefficient of variation.

*3.4.2 Voxelwise SUVs (60.5 – 90.5 minutes)*

The variant yielded inconsistent data for most ROIs, with high to very high MA-TDs (≥ 15%) in all non-reference ROIs other than the inferior frontal gyrus (Table 10). ICCs ranged from 0.62 to 0.75, with a median (iqr) of 0.67 (0.64 – 0.71).

|  | **Median**  **SUV** | **iqr** | **Min** | **Max** | **Median**  **%**  **WS-CV** | **Median signed**  **% t-rt diff** | **% t-rt diff range** | **MA-TD**  **(%)** | **Mean %**  **BS-CV** | **ICC** |
| --- | --- | --- | --- | --- | --- | --- | --- | --- | --- | --- |
| **ACG** | 2.13 | 1.93 – 2.54 | 1.71 | 3.04 | 27 | 21 | -10 – 24 | 21 | 20 | 0.62 |
| **Fusiform gyrus** | 2.23 | 1.93 – 2.47 | 1.80 | 2.99 | 33 | 15 | -1 – 26 | 15 | 19 | 0.65 |
| **Hippocampus** | 2.71 | 2.34 – 3.20 | 2.20 | 3.45 | 23 | 16 | -8 – 18 | 16 | 18 | 0.72 |
| **Inferior frontal gyrus** | 1.49 | 1.31 – 1.85 | 1.14 | 2.11 | 28 | 12 | -14 – 23 | 14 | 22 | 0.75 |
| **Insula** | 2.26 | 1.91 – 2.65 | 1.81 | 2.93 | 26 | 20 | -10 – 21 | 20 | 18 | 0.63 |
| **Occipital lobes** | 1.07 | 0.95 – 1.32 | 0.85 | 1.54 | 34 | 17 | -9 – 24 | 17 | 21 | 0.68 |
| Median (iqr): |  |  |  |  | **28**  **(26 – 32)** |  |  | **17**  **(15 – 19)** | **20**  **(18 – 21)** | **0.67 (0.64 – 0.71)** |
|  |  |  |  |  |  |  |  |  |  |  |
| **Brainstem** | 0.29 | 0.28 – 0.34 | 0.25 | 0.47 | 163 | 14 | -19 – 25 | 16 | 23 | 0.70 |
| **Cerebellum** | 0.57 | 0.54 – 0.69 | 0.47 | 0.86 | 100 | 15 | -9 – 27 | 15 | 21 | 0.69 |

*Table 10 Participants’ SUVs (voxelwise, 60.5 – 90.5 minutes).*  ACG – anterior cingulate gyrus, diff – difference, BS-CV – between-subject coefficient of variation, ICC – intraclass correlation coefficient, iqr – interquartile range, MA-TD – median absolute test – retest difference, Max – maximum, Min – minimum, SUV – standardised uptake value, t-rt – test – retest, WS-CV – within-subject coefficient of variation.

*3.4.3 SRTM using brainstem*

The method yielded very low to low MA-TDs for all ROIs (≤ 8%; Table 11). ICCs ranged from -0.33 to 0.77, with a median (iqr) of 0.34 (0.22 – 0.59). Quantification in the hippocampus was very reproducible and moderately reliable (MA-TD 3%, ICC 0.77).

|  | **Median**  **BP_ND_** | **iqr** | **Min** | **Max** | **Median RSS** | **Median signed**  **% t-rt diff** | **% t-rt diff range** | **MA-TD (%)** | **Mean %**  **BS-CV** | **ICC** |
| --- | --- | --- | --- | --- | --- | --- | --- | --- | --- | --- |
| **ACG** | 4.37 | 4.20 – 4.51 | 4.03 | 4.75 | 2.04 | 5 | -2 – 17 | 5 | 5 | -0.33 |
| **Fusiform gyrus** | 4.05 | 3.88 – 4.30 | 3.71 | 4.44 | 1.07 | 8 | -1 – 13 | 8 | 5 | 0.22 |
| **Hippocampus** | 4.73 | 4.45 – 5.10 | 4.31 | 5.31 | 1.47 | 2 | -3 – 11 | 3 | 8 | 0.77 |
| **Inferior frontal gyrus** | 3.19 | 3.05 – 3.43 | 2.87 | 3.58 | 2.47 | 1 | -5 – 12 | 5 | 8 | 0.63 |
| **Insula** | 4.25 | 4.10 – 4.50 | 3.93 | 4.73 | 2.20 | 7 | -3 – 14 | 7 | 6 | 0.22 |
| **Occipital lobes** | 2.22 | 2.16 – 2.37 | 2.07 | 2.61 | 2.15 | 4 | -2 – 14 | 4 | 7 | 0.46 |
| Median (iqr): |  |  |  |  | **2.10**  **(1.61 – 2.19)** |  |  | **5**  **(4 – 7)** | **7**  **(5 – 8)** | **0.34 (0.22 – 0.59)** |
|  |  |  |  |  |  |  |  |  |  |  |
| **Brainstem** | -- | -- | -- | -- | -- | -- | -- | -- | -- | -- |
| **Cerebellum** | 0.96 | 0.93 – 1.01 | 0.83 | 1.25 | 1.48 | -5 | -14 – 21 | 14 | 10 | 0.21 |

*Table 11 Participants’ BP_ND_ (regional SRTM using brainstem).* ACG – anterior cingulate gyrus, BP_ND_ – binding potential relative to non-displaceable binding, diff – difference, BS-CV – between-subject coefficient of variation, ICC – intraclass correlation coefficient, iqr – interquartile range, MA-TD – median absolute test – retest difference, Max – maximum, Min – minimum, RSS – residual sum of squares, SRTM – simplified reference tissue model, t-rt – test – retest.

*3.4.4 Voxelwise SRTM2 using brainstem*

This variant yields parametric images of BP_ND_; an example is provided in manuscript proper, Figure 4. The variant yielded consistent data for all ROIs, with very low to low MA-TDs (≤ 6%; Table 12). ICCs ranged from -0.18 to 0.85, with a median (iqr) of 0.42 (0.29 – 0.62). Quantification in the hippocampus was very reproducible and very reliable (MA-TD 2%, ICC 0.85).

|  | **Median**  **BP_ND_** | **iqr** | **Min** | **Max** | **Median %**  **WS-CV** | **Median signed**  **% t-rt diff** | **% t-rt diff range** | **MA-TD (%)** | **Mean %**  **BS-CV** | **ICC** |
| --- | --- | --- | --- | --- | --- | --- | --- | --- | --- | --- |
| **ACG** | 4.38 | 4.16 – 4.58 | 3.98 | 4.69 | 25 | 6 | 0 – 16 | 6 | 5 | -0.18 |
| **Fusiform gyrus** | 4.06 | 3.84 – 4.33 | 3.64 | 4.43 | 35 | 6 | 3 – 15 | 6 | 6 | 0.27 |
| **Hippocampus** | 4.49 | 4.19 – 4.94 | 4.08 | 5.04 | 22 | -1 | -2 – 11 | 2 | 9 | 0.85 |
| **Inferior frontal gyrus** | 3.25 | 3.09 – 3.48 | 2.90 | 3.61 | 23 | 3 | -6 – 12 | 5 | 8 | 0.66 |
| **Insula** | 4.24 | 4.03 – 4.46 | 3.88 | 4.66 | 23 | 6 | -2 – 15 | 6 | 6 | 0.34 |
| **Occipital lobes** | 2.33 | 2.29 – 2.49 | 2.25 | 2.71 | 26 | 1 | 0 – 13 | 1 | 7 | 0.50 |
| Median (iqr): |  |  |  |  | **24**  **(23 – 26)** |  |  | **6**  **(3 – 6)** | **7**  **(6 – 8)** | **0.42 (0.29 – 0.62)** |
|  |  |  |  |  |  |  |  |  |  |  |
| **Brainstem** | -- | -- | -- | -- | -- | -- | -- | -- | -- | -- |
| **Cerebellum** | 1.11 | 1.06 – 1.19 | 0.99 | 1.42 | 50 | -2 | -9 – 20 | 9 | 10 | 0.40 |

*Table 12 Participants’ BP_ND_ (voxelwise SRTM2 using brainstem).* ACG – anterior cingulate gyrus, BP_ND_ – binding potential relative to non-displaceable binding, diff – difference, BS-CV – between-subject coefficient of variation, ICC – intraclass correlation coefficient, iqr – interquartile range, MA-TD – median absolute test – retest difference, Max – maximum, Min – minimum, SRTM – simplified reference tissue model, t-rt – test – retest, WS-CV – within-subject coefficient of variation.

*3.4.5 Regional SRTM using cerebellum.*

The variant yielded consistent data for all ROIs, with very low to low MA-TDs (≤ 5%; Table 13). ICCs ranged from 0.59 to 0.95, with a median (iqr) of 0.71 (0.64 – 0.84). Quantification in the hippocampus was very reproducible and very reliable (MA-TD 3%, ICC 0.95).

|  | **Median**  **BP_ND_** | **iqr** | **Min** | **Max** | **Median RSS** | **Median signed**  **% t-rt diff** | **% t-rt diff range** | **MA-TD (%)** | **Mean %**  **BS-CV** | **ICC** |
| --- | --- | --- | --- | --- | --- | --- | --- | --- | --- | --- |
| **ACG** | 1.66 | 1.61 – 1.74 | 1.55 | 1.98 | 1.09 | 2 | -1 – 14 | 2 | 8 | 0.59 |
| **Fusiform gyrus** | 1.54 | 1.48 – 1.60 | 1.41 | 1.78 | 0.74 | 2 | -5 – 11 | 5 | 7 | 0.68 |
| **Hippocampus** | 1.90 | 1.83 – 2.11 | 1.80 | 2.20 | 0.77 | -1 | -4 – 3 | 3 | 8 | 0.95 |
| **Inferior frontal gyrus** | 1.11 | 1.00 – 1.16 | 0.96 | 1.21 | 1.10 | -1 | -5 – 7 | 5 | 9 | 0.87 |
| **Insula** | 1.68 | 1.57 – 1.71 | 1.51 | 1.90 | 0.88 | 3 | -1 – 10 | 3 | 7 | 0.74 |
| **Occipital lobes** | 0.61 | 0.58 – 0.65 | 0.54 | 0.70 | 1.08 | 2 | -5 – 16 | 5 | 9 | 0.63 |
| Median (iqr): |  |  |  |  | **0.98**  **(0.80 – 1.09)** |  |  | **4**  **(3 – 5)** | **8**  **(7 – 9)** | **0.71 (0.64 – 0.84)** |
|  |  |  |  |  |  |  |  |  |  |  |
| **Brainstem** | -- | -- | -- | -- | -- | -- | -- | -- | -- | -- |
| **Cerebellum** | -- | -- | -- | -- | -- | -- | -- | -- | -- | -- |

*Table 13 Participants’ BP_ND_ (regional SRTM using cerebellum).* ACG – anterior cingulate gyrus, BP_ND_ – binding potential relative to non-displaceable binding, diff – difference, BS-CV – between-subject coefficient of variation, ICC – intraclass correlation coefficient, iqr – interquartile range, MA-TD – median absolute test – retest difference, Max – maximum, Min – minimum, RSS – residual sum of squares, SRTM – simplified reference tissue model, t-rt – test – retest.

*3.4.6 Voxelwise SRTM2 using cerebellum*

This variant yields parametric images of BP_ND_; an example is provided in the manuscript proper, Figure 5. The variant yielded consistent data for all ROIs, with very low MA-TDs (< 5%; Table 14). ICCs ranged from 0.64 to 0.93, with a median (iqr) of 0.83 (0.70 – 0.86). Quantification in the hippocampus was very reproducible and very reliable (MA-TD 2%, ICC 0.93).

|  | **Median**  **BP_ND_** | **iqr** | **Min** | **Max** | **Median**  **%**  **WS-CV** | **Median signed**  **% t-rt diff** | **% diff range** | **MA-TD**  **(%)** | **Mean %**  **BS-CV** | **ICC** |
| --- | --- | --- | --- | --- | --- | --- | --- | --- | --- | --- |
| **ACG** | 1.64 | 1.56 – 1.71 | 1.53 | 1.97 | 33 | 1 | -1 – 12 | 1 | 8 | 0.66 |
| **Fusiform gyrus** | 1.56 | 1.47 – 1.59 | 1.35 | 1.77 | 47 | 4 | -3 – 12 | 4 | 8 | 0.64 |
| **Hippocampus** | 1.78 | 1.64 – 1.99 | 1.55 | 2.08 | 30 | -2 | -8 – 1 | 2 | 11 | 0.93 |
| **Inferior frontal gyrus** | 1.12 | 0.99 – 1.17 | 0.95 | 1.22 | 35 | -3 | -5 – 8 | 4 | 10 | 0.87 |
| **Insula** | 1.66 | 1.49 – 1.69 | 1.45 | 1.84 | 31 | 1 | -2 – 9 | 2 | 8 | 0.84 |
| **Occipital lobes** | 0.64 | 0.63 – 0.68 | 0.58 | 0.74 | 46 | 2 | -3 – 9 | 3 | 9 | 0.81 |
| Median (iqr): |  |  |  |  | **34**  **(32 – 43)** |  |  | **3**  **(2 – 4)** | **9**  **(8 – 10)** | **0.83**  **(0.70 – 0.86)** |
|  |  |  |  |  |  |  |  |  |  |  |
| **Brainstem** | -- | -- | -- | -- | -- | -- | -- | -- | -- | -- |
| **Cerebellum** | -- | -- | -- | -- | -- | -- | -- | -- | -- | -- |

*Table 14 Participants’ BP_ND_ (voxelwise SRTM2 using cerebellum).* ACG – anterior cingulate gyrus, BP_ND_ – binding potential relative to non-displaceable binding; diff – difference, BS-CV – between-subject coefficient of variation, ICC – intraclass correlation coefficient, iqr – interquartile range, MA-TD – median absolute test – retest difference, Max – maximum, Min – minimum, SRTM – simplified reference tissue model, t-rt – test – retest, WS-CV – within-subject coefficient of variation.

# 4. References

Hammers, A., Asselin, M.C., Turkheimer, F.E., Hinz, R., Osman, S., Hotton, G., Brooks, D.J., Duncan, J.S., Koepp, M.J., 2007. Balancing bias, reliability, noise properties and the need for parametric maps in quantitative ligand PET: [^11^C]diprenorphine test-retest data. NeuroImage 38, 82-94.

Riaño Barros, D.A., McGinnity, C.J., Rosso, L., Heckemann, R.A., Howes, O.D., Brooks, D.J., Duncan, J.S., Turkheimer, F.E., Koepp, M.J., Hammers, A., 2014. Test–retest reproducibility of cannabinoid-receptor type 1 availability quantified with the PET ligand [^11^C]MePPEP. NeuroImage 97, 151-162.
